# Towards linked open gene mutations data

**DOI:** 10.1186/1471-2105-13-S4-S7

**Published:** 2012-03-28

**Authors:** Achille Zappa, Andrea Splendiani, Paolo Romano

**Affiliations:** 1 Bioinformatics, IRCCS AOU San Martino-IST National Cancer Research Institute, Genoa, I-16132, Italy; 2 Department of Informatics, Systems and Telematics, University of Genoa, Genoa, I-16145, Italy; 3 Rothamsted Research, West Common, Harpenden, Hertfordshire, AL5 2JQ, UK; 4 Digital Enterprise Research Institute, National University of Ireland at Galway, IDA Business Park, Lower Dangan, Galway, Ireland

## Abstract

**Background:**

With the advent of high-throughput technologies, a great wealth of variation data is being produced. Such information may constitute the basis for correlation analyses between genotypes and phenotypes and, in the future, for personalized medicine. Several databases on gene variation exist, but this kind of information is still scarce in the Semantic Web framework.

In this paper, we discuss issues related to the integration of mutation data in the Linked Open Data infrastructure, part of the Semantic Web framework. We present the development of a mapping from the IARC TP53 Mutation database to RDF and the implementation of servers publishing this data.

**Methods:**

A version of the IARC TP53 Mutation database implemented in a relational database was used as first test set. Automatic mappings to RDF were first created by using D2RQ and later manually refined by introducing concepts and properties from domain vocabularies and ontologies, as well as links to Linked Open Data implementations of various systems of biomedical interest.

Since D2RQ query performances are lower than those that can be achieved by using an RDF archive, generated data was also loaded into a dedicated system based on tools from the Jena software suite.

**Results:**

We have implemented a D2RQ Server for TP53 mutation data, providing data on a subset of the IARC database, including gene variations, somatic mutations, and bibliographic references. The server allows to browse the RDF graph by using links both between classes and to external systems. An alternative interface offers improved performances for SPARQL queries. The resulting data can be explored by using any Semantic Web browser or application.

**Conclusions:**

This has been the first case of a mutation database exposed as Linked Data. A revised version of our prototype, including further concepts and IARC TP53 Mutation database data sets, is under development.

The publication of variation information as Linked Data opens new perspectives: the exploitation of SPARQL searches on mutation data and other biological databases may support data retrieval which is presently not possible. Moreover, reasoning on integrated variation data may support discoveries towards personalized medicine.

## Background

### The conversion of relational database contents into the Semantic Web

The Semantic Web [1] is gaining momentum as a framework for the development of next-generation bioinformatics data integration tools since its standards and technologies seem to have now reached enough maturity to be considered a viable solution for data integration challenges. Semantic Web based approaches to biomedical data integration have already been proposed a number of times in recent years [2-6]. Most recently an approach known as Linked Data, or Web of Data is being explored.

The vision of the Semantic Web is to evolve the Web into a distributed knowledge base: this vision relies on its evolution from the current Web of Documents, where each node of the network is represented by an unstructured document, into a Web of Data, where each node represents machine processable information. In this context, access to information is achieved through portals and search engines whose behavior is supported by semantic features. A good introduction to the Web of Data can be found in [7].

A relevant contribution to this evolution of the Web may come from the conversion of data stored in Relational Databases (RDB) into a viable representation such as the Resource Description Framework (RDF) [8], which is the basic technology to represent information in the Semantic Web. RDF is based on the composition of simple predicates ("triples") made by three elements identifying "Subject", "Predicate" (or "Property"), and "Object". Here, semantics can easily be associated to property definitions, while subjects usually are well identified entities and objects may either represent related entities or values.

Many research works have therefore been focused either on the static conversion or on the dynamic mapping of data from RDB to RDF. They have led to the implementation of both mapping tools and domain-specific applications. Some mappings are automatically generated via a simple association where the name of the relational table is mapped to an RDF class node and the names of its columns are used as RDF predicates. As a consequence, cell values are mapped to instances or data values. In this case, entities and relations, as well as their meaning, reflect the RDB schema and the knowledge of the schema is needed to understand the exported information.

In other mappings, relations and entities of the original databases are converted to a representation which is instead based on a shared conceptualization that can be, even significantly, different from the schema of the database. Differences may relate to properties, relationships, and even entity values (e.g., different coding applied, split/merged values). In this case, automatic mappings can serve as a starting point to quickly create customized, domain-specific mappings.

Relational to RDF mapping software exists both as independent tools (e.g.: D2RQ and Triplify), or as part of a larger suite (e.g. Allegrograph, Sesame, OWLim, Virtuoso). In general, they are components of a wider range of software solutions which can expose RDF entities and relations in structured information resources. A list of these tools is available on-line [9].

In the biomedical domain, an exemplar resource is represented by Bio2RDF [10], a system that allows an integrated access to a vast number of biomedical databases through Semantic Web technologies, i.e. RDF for data representation and SPARQL (SPARQL Protocol and RDF Query Language) [11] for queries. To this aim, many databases have been converted to RDF by special scripts, called RDFizers, while some information systems that were already offering a viable format and interface where directly linked to the system.

This conversion was based on a unified ontology, taking care of properties included in the information resources already available in RDF. Moreover, the system provided a unified URI schema, overcoming heterogeneity of URIs already provided by other systems. All major genomics, proteomics, networks and pathways, and nomenclatures databases were included in the system, as well as some clinical, e.g. Online Mendelian Inheritance in Man (OMIM), and bibliographic ones, e.g. PubMed, and the Gene Ontology.

The Linked Open Data (LOD) initiative, a Community Project at World Wide Web Consortium (W3C), aims at extending "the Web with a data commons by publishing various open data sets as RDF on the Web and by setting RDF links between data items from different data sources" [12]. In this context, many biomedical databases have already been made available (a Linked Open Data cloud diagram is available on-line [13]). Many of these datasets derive from Bio2RDF, but there are also some that were independently built, e.g. Diseasome, a dataset extracted from OMIM that includes information on disorders and disease-related genes linked by known associations.

### Human variation data and the Semantic Web

In the last decade, with the advent of high-throughput technologies, sequencing has become faster and less expensive. As a consequence, a great wealth of data is being produced in order to identify variation data, i.e. specific, individual and sub-population related information. One of the best known projects of this kind is "1,000 Genomes", an international collaboration that recently ended its pilot phase [14,15]. The goal of the pilot phase was the identification of at least the 95% of variations present in at least 1% of individuals in three distinct populations by means of Next-Generation Sequencing technologies. This led to the production of ca. 4.9 Tbases (about 3 Gbases/individual) and to the determination of 15 millions mutations, 1 million deletions/insertions, and 20,000 variants of greater size.

Such information constitutes the basis on which genomics may meet clinical information, correlation analyses between genotypes and phenotypes may be carried out, and the perspectives of genomic or personalized medicine may be realized [16,17].

Although several databases on gene mutation and variation for humans exist, their semantic annotation is very limited and their formats are heterogeneous. Overall, only a little information on human variation is included in the Web of Data and/or it is available on-line in implementations that are based on Semantic Web technologies. This is the case, e.g., for the data on impact of protein mutations on their function that was extracted from scientific literature by using a specialized text mining pipeline by Laurila et al [18] (in this case, data is available on-line through a SPARQL endpoint, but access is restricted to authorized users only).

Lists of Locus Specific Data Bases (LSDB) and other databases related to human variation, like those related to Disease Centered Mutations, SNPs (Single Nucleotide Polymorphisms), National and Ethnic Mutations, Mitochondrial Mutations, and Chromosomal Variation, are available on-line at the site of the Human Genome Variation Society (HGVS) [19,20], although many of these lists are not up-to-date. Indeed, the best human variation information is available in curated databases, many of which are managed by means of the Leiden Open Variation Database (LOVD) [21] schema and system. Many other databases are managed by proprietary systems. The Human Variome Project (HVP) [22] has produced recommendations for nomenclatures of variations and for contents of mutation databases.

The issue of integrating variation data with molecular biology databases is however well known. Conditions for the integration of LSDBs with other biological databases have been outlined by den Dunnen et al in [23]. In this paper, a distinction is made between the information that should be shared and the one that could be shared. In the former set, only some reference data are defined, including contact information for the database, identifiers of the gene in various databases, a unique reference to the sequence, and the description of the mutation at DNA level. In the latter set, that includes data on original bibliography, changes at protein and RNA levels, and associated pathogeniticy, issues related to ownerships and quality of data are also present.

### Shared property definitions for human variation data

Integrating data on the Semantic Web is mainly a matter of shared and reusable properties' definitions and unique data identifiers. Some mutation related ontologies exist. These include the Variation Ontology (VariO) [24] and the Mutation Impact Ontology (MIO) [25].

VariO is still in a development phase, not officially released for annotation or analysis purposes. It is aiming at providing standardized, systematic descriptions of effects and consequences of position specific variations. It can be used to describe effects and consequences of variations at different levels (DNA, RNA protein). VariO reuses some terms and definitions from Sequence Ontology (SO), Gene Ontology (GO), and other ontologies.

MIO was developed to support semantic extraction and grounding of mutation impact data from literature. The ontology has a strong use case in the publication of text mining results through semantic Web Services [18] in the framework of the Semantic Automated Discovery and Integration (SADI) [26,27] infrastructure.

Other biological ontologies making reference to mutations also exist, such as the Sequence Ontology (for a recent assessment of the state and issues in incorporating mutation information in SO see [28]). However, a specific ontology able to represent or support representation of gene variation data is not available yet.

Even more relevant, a specific framework for identifying variations is missing. HGVS nomenclature defines mutations in relation to a specific version of RefSeq, which leaves the reconciliation of mutations described with reference to different RefSeq versions problematic. In a LOD framework, this is a key issue as having common URIs for the same mutations is a key for the integration of different datasets.

Furthermore, the definition of equivalent mutation relies on an abstraction which is based on sequence similarity. As such, it is not easily deducible by using common inference mechanisms which are based on Semantic Web technologies and tools (e.g.: a cluster of sequences may *de facto* inform a class which is characterized by the related consensus sequence).

Solutions which incorporate services in the LOD, e.g. SADI, may provide a unified framework where ontology languages and sequence alignment services could be used to compute the equivalence of mutations.

### Aim of this work

In this paper, we cope with issues related to the integration of mutation data in the Linked Open Data infrastructure. We present the development of a mapping between a relational version of the IARC TP53 Mutation database (IARCDB) to RDF that takes into account HGVS recommendations as well as existing ontologies for the representation of this domain knowledge. A first implementation of servers publishing this data in RDF with the aim of studying issues related to the integration of mutation data in the Linked Open Data cloud is also presented.

## Methods

### Software infrastructure

Linked Data is an approach to publish information on the web which eases the integration of data from different sources by relying both on RDF as a *lingua franca *for a machine processable representation of information and on shared ontologies in order to allow the information from different resources to be semantically connected to each other. RDF itself however does not provide domain-specific terms. These may be identified by adopting shared taxonomies, vocabularies, and ontologies. Suitable terms in existing ontologies should of course be reused whenever possible: new terms should only be added when a viable term does not exist. RDF properties must then be mapped to external ontologies, while resources (objects and subjects in RDF triples) must be linked to LOD repositories by using shared identifiers, and comments and definitions must be added whenever possible.

By using dereferenceable URIs, i.e. URIs for which it is possible to get information about the referenced resource on the Web, as global identifiers for resources, linked data makes it possible to set hyperlinks between entities in different data sources. Such links are the glue connecting data islands of the Web of Data into a global, interconnected data space.

In our case, automatic RDB to RDF mappings were first created by using D2RQ, a platform for treating relational databases as virtual RDF graphs [29]. This tool allows on-the-fly generation of RDF triples from a database. It also allows browsing the generated RDF triples through a standard web interface and querying the relational database through a SPARQL endpoint.

The query performance of D2RQ is lower than that which can be achieved by using a devoted RDF triple store. In order to evaluate reliability and performances of an on-the-fly mapping system, such as D2RQ, compared to a native RDF framework, a dump of all triples generated by D2RQ was loaded into a dedicated Jena TDB triple store [30], a SPARQL Database for Jena [31] that provides for large scale storage and querying of RDF datasets. Many triple stores exist which varies in features and performance. We have opted for Jena as it was providing sufficient performance and, at the same time, a good set of integrated and interoperable tools which was ideal for our prototype lead development approach.

We have then enriched our dataset by adding triples connecting samples to related UMLS concepts. This mapping was implemented by means of a SPARQL Update federated query interconnecting our dataset with the Linked Life Data (LLD) endpoint.

Once the RDF dataset was created, it was made accessible as a SPARQL endpoint using Joseki [32], a Jena tool that provides support for SPARQL queries through an HTTP engine. Joseki was configured to connect to the TDB database and it was connected to an implementation of SPARQLer [31], a user friendly interface to a SPARQL server. Finally, we exposed the content of our TDB triple store as a Linked Data interface using Pubby, a well known Linked Data frontend for SPARQL endpoints [33].

### Datasets

The IARC TP53 Database has been maintained at the International Agency for Research on Cancer (IARC) in Lyon, France, since 1994 [34]. The database compiles all TP53 mutations that have been reported in the published literature since 1989 [35]. It includes annotations on functional impact of mutations, either predicted or experimentally assessed, clinico-pathologic characteristics of tumors and demographic and life-style information on patients.

Various datasets, corresponding to different views of the available data, are made available to interested users as spreadsheets for download. The relational schema, however, is not public. On-line queries are meant to allow for data analysis only, answering to such questions as "Search for TP53 mutation prevalence in selected types of tumor and populations", and "Display a histogram showing the distribution of tumors associated with the selected germline or somatic mutation(s)".

For many years, in the sphere of a collaboration between IARC and the National Cancer Research Institute of Genova, datasets have been implemented in a relational databases management system at IST as a basis for an SRS implementation of the IARC TP53 Mutation Database [36,37].

### Development of mappings

The first mapping file was produced automatically by D2RQ. It was then manually refined to improve its commitment to a shared representation and, thus, to encode in the mapping some semantics that is not expressed in the RDB schema.

For instance, D2RQ generates predicate names which are based on the RDB column names: there is no way to know when a predicate refers to a property for which a shared representation exists. By customizing predicates we have been able to better represent the semantics of our data, according to shared ontologies.

Only a limited set of external ontologies and terminologies have been taken into account. These include the NCI Thesaurus (NCIT) [38,39] for medical terminology (namely topography and morphology), the Bibliographic Ontology (BIBO) [40] and the BibTeX definition in Web Ontology Language (OWL) [41] for bibliographic references, the Diseasome ontology [42,43] and MIO [25].

Moreover, external links were set to LOD implementations of DBpedia, a system including all structured information which is present in Wikipedia pages [44], PubMed, the Human Genome Nomenclature Committee (HGNC) database [45], the On-line Mendelian Inheritance in Man (OMIM) system, UniProt, and the Unified Medical Language System (UMLS). All links were defined to the Bio2RDF entry points of these databases, expressed by using the unified Bio2RDF URI style, with the exception of DBpedia, that was linked through its own namespace, and UMLS, that was connected through LinkedLifeData [46].

We also made reference to some other frequently used vocabularies such as rdf:, rdfs:, and owl:. Where shared relations were not available to express the content of our database, we have used ad-hoc defined properties. The re-use of ontologies is not limited to relations. The majority of "values" in the IARC database comes from controlled vocabularies and reference dictionaries and therefore they are *de facto* ontology terms. We have mapped these terms to classes from ontologies (or terminologies) such as UMLS.

URIs (identifiers of nodes in RDF) have been made compatible, where possible, with Bio2RDF and PubMed. On the whole, these implementation choices allow us to state that our system is deployed according to Linked Data principles [47]. Three examples of mappings are reported in table 1. The first example shows the association of the title of a paper to an entity representing the related bibliographic reference through the bibtex:hasTitle property. The second one presents the association of the wild type aminoacid to the corresponding gene variation through the mio:hasWildTypeResidue property. The last one connects our implementation with an external entity, namely the Bio2RDF implementation of the HGNC database.

**Table 1:** Examples of D2RQ mappings

Description	Mapping	Generated triple (example)
Create a triple that defines the value of the title of a given bibliographic reference.	map:somatic_ref_Title a d2rq:PropertyBridge; d2rq:belongsToClassMap map:somatic_ref; d2rq:property bibtex:hasTitle; d2rq:column "SomaticRef15.Title";.	<http://bioinformatics.istge.it/logvd/resource/somatic_refs/7> <http://data.bibbase.org/ontology/#hasTitle> "Mutations in the p53 gene occur in diverse human tumour types"

Create a triple that defines the value of the wild type aminoacid for a given gene variation. Conditional clauses avoid definitions for special cases (empty field, dash character and "NA" value).	map:variation_hasWildTypeResidue a d2rq:PropertyBridge; d2rq:belongsToClassMap map:variation; d2rq:property mio:hasWildTypeResidue; d2rq:column "mutations15.WT_AA"; d2rq:condition "mutations15.WT_AA ! = ('NA')"; d2rq:condition "mutations15.WT_AA ! = ('')"; d2rq:condition "mutations15.WT_AA ! = ('-')";.	<http://bioinformatics.istge.it/logvd/resource/variations/994> <http://unbsj.biordf.net/ontologies/mutation-impact-ontology.owl#hasWildTypeResidue>"P"

Creates a triple that establishes a link to the TP53 human gene description in HGNC as implemented in Bio2RDF.	map:gene_HGNC a d2rq:PropertyBridge; d2rq:belongsToClassMap map:gene; d2rq:property diseasome:hgncId; d2rq:uriPattern "http://bio2rdf.org/hgnc:11998";.	<http://bioinformatics.istge.it/logvd/resource/genes/TP53> <http://www4.wiwiss.fu-berlin.de/diseasome/resource/diseasome/hgncId> <http://bio2rdf.org/hgnc:11998>

Three examples of D2RQ mappings between the IARC TP53 Mutation database and its RDF representation. In the first one, the title of a paper is just extracted from the database and associated to an entity representing a given bibliographic reference by means of the bibtex:hasTitle property. In the second example, the one-letter code of the wild type aminoacid corresponding to the location of a given variation is associated to the entity representing the same variation through the mio:hasWildTypeResidue property. The third example creates a connection with an external entity, namely the HGNC identifier of the TP53 gene, by specifying its Linked Data URI.

The Additional file 1 includes the complete mapping.

## Results

### A prototype implementation

We have implemented a D2RQ Server for TP53 mutation data as a prototype for studying issues related to the publication of mutation data on the LOD framework. It provides data on a significant subset of the IARC TP53 database, including gene variations, somatic mutations, and related bibliographic references. In order to minimize duplications, information on samples and individuals have been made available separately from the related mutations.

**Figure 1:**
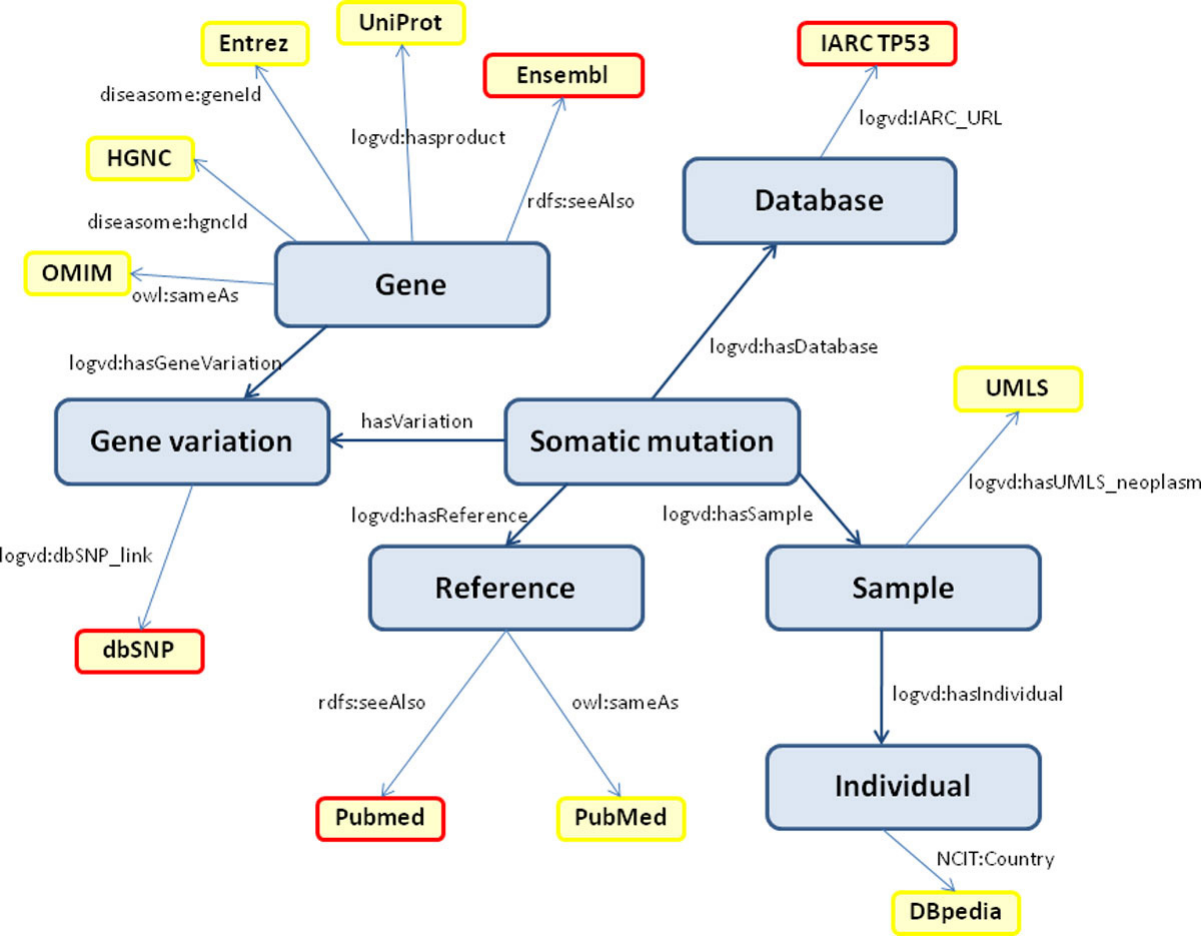
**Classes, relationships and external links**. A schematic representation of classes that were created by the mapping, of their relationships, and of external links is presented in this figure. The great boxes represent classes, while the smaller represent external datasets. In the latter case, a yellow border denotes RDF dataset linked by URIs, a red one denotes web sites linked by URLs.

In table 2, we present a summary of published classes and the correspondence with the original datasets. In Figure 1, the schematic representation of classes that were created by the mapping, of their relationships, and of external links is presented.

**Table 2:** Correspondence between classes and original datasets

Class	Description	Linked to	IARC datasets
database	Database information	somatic_mutation	Implicit

gene	Gene information	gene_variation	Implicit

gene_variation	Detailed description of the mutation	gene, somatic_mutation	Gene variations

somatic_mutation	Summary mutation data, linked to bibliography, sample, and variation data	database, sample, gene_variation, somatic_ref	Somatic mutations

sample	Tumor topography, morphology, origin, and classification	somatic_mutation, individual	Somatic mutations

individual	Demographic details, life-style data and genetics of the donor	sample	Somatic mutations

somatic_ref	Bibliographic references where mutations are described	somatic_mutation	Somatic references

A summary of published classes and the correspondence with the original datasets.

**Figure 2:**
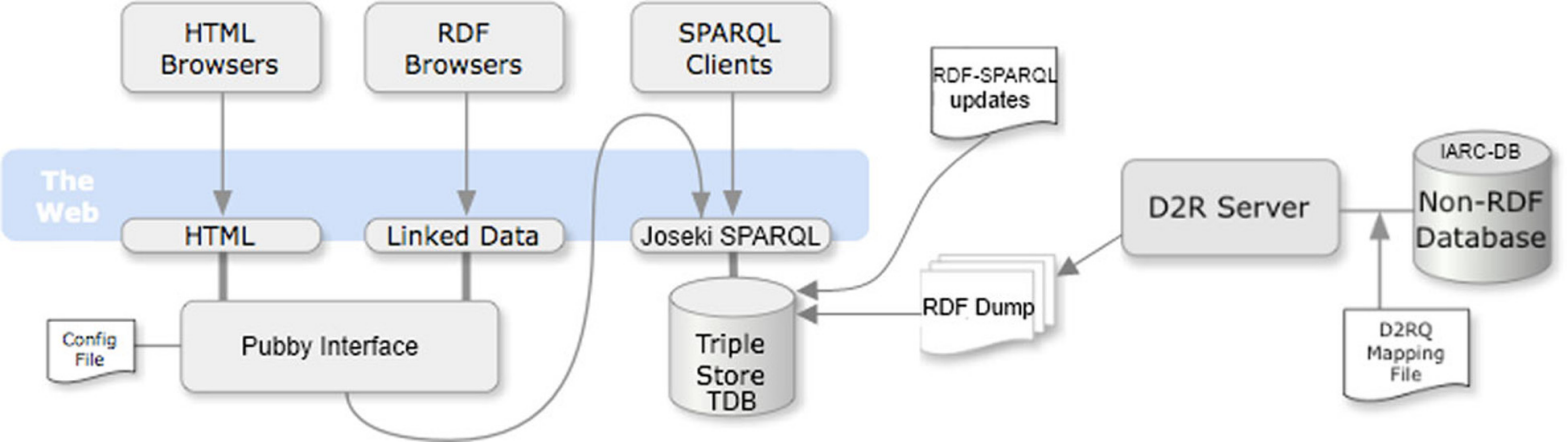
**Architecture of the system**. The components of the system and their interfaces are shown. The triple store is populated by the RDF dump, that is created by D2RQ, and incremented by special SPAQRL updates. Access to the triple store is granted through Joseki, which can be queried by SPARQL clients. The Pubby interface allows data navigation by means of both HTML and RDF browsers.

In Figure 2, the architecture of the overall system is shown. In brief, the original relational database is translated in RDF and exposed using the D2RQ framework. A RDF dump is stored into a TDB triple store that can be queried through a Joseki SPARQL endpoint either directly (through some SPARQL client) or by means of the Pubby interface that in turn may be queried by both HTML and RDF browsers.

### Providing access to the information

**Figure 3:**
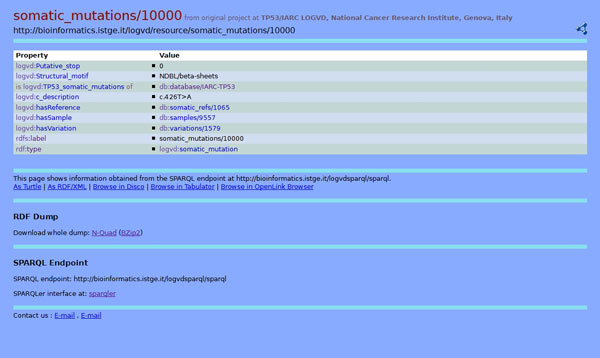
**Browsing the RDF graph: a somatic mutation**. Representation of properties and values of a defined somatic mutation (somatic_mutations/10000), including links to related gene variation (variations/1579), sample (samples/9557), and bibliographic reference (somatic_refs/1065), together with some proper attributes (mutation and structural motif). This class is central within the schema, linking the majority of classes.

As previously reported, the Pubby server gives access to the information through various interfaces. It allows browsing the RDF graph starting from any page: further navigation of the graph is achieved by internal links. E.g., one can select a defined somatic mutation (somatic_mutations/10000) and see links to related gene variation (variations/1579), sample (samples/9557), and bibliographic reference (somatic_refs/1065), along with some proper attributes, like the mutation (c.426T > A) or structural motif (NDBL/beta-sheets). See Figure 3.

**Figure 4:**
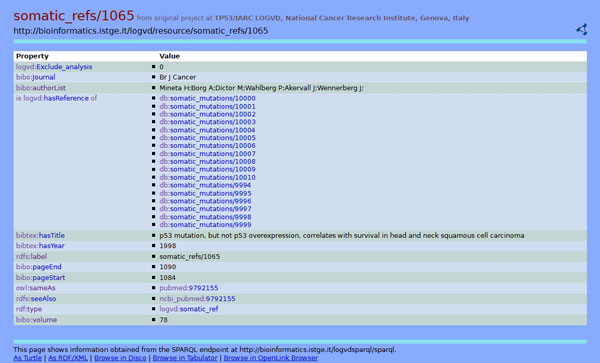
**Browsing the RDF graph: a bibliographic reference**. Properties and objects associated to the bibliographic reference denoted by somatic_refs/1065 are shown. All links to somatic references described in the paper are presented. Links to Pubmed referring to its Bio2RDF implementation and to the NCBI web site are also shown.

Of course, this way of browsing the RDF graph allows to reach further pages. E.g., in the previous case, from a single mutation one can reach the triples associated to the related bibliographic reference and thus have a list of all mutations that were described in the same paper. In Figure 4 all properties and objects associated to the bibliographic reference denoted by somatic_refs/1065 are shown. In this figure, links to all somatic mutations described in the paper are presented. Moreover, two links to Pubmed are shown: the first one refers to its implementation in Bio2RDF and allows to extend the navigation of the virtual RDF graph externally from our implementation, while the second one relates to the interface that is usually accessed by researchers at NCBI.

More precisely, Pubby is used to add a Linked Data interface to our SPARQL endpoint. It handles external requests by connecting to the SPARQL endpoint, issuing a SPARQL "DESCRIBE" query about the requested URI, and showing the result in a HTML or RDF page, supporting Linked Data compliant content resolution and negotiation procedures.

An additional interface to the SPARQL endpoint is provided via SPARQLer, a simple interface to perform SPARQL queries. In this case, all prefixes that are needed to properly identify RDF nodes are added by the system, so that the compilation of the query is simplified.

Moreover, the RDF data set that we implemented can be explored by using any Semantic Web browser or application, like Marbles [48]. The SPARQL endpoint can also be queried by using some more sophisticated tool, such as RelFinder [49].

### Some example queries

With the aim to show which kind of questions can currently be posed to our implementation, we are presenting here three example queries that can be carried out through the TDB-Joseki SPARQL endpoint [50]. A summary of these queries and of related results is shown in Tables 3, 4, and 5.

**Table 3:** SPARQL query example 1: descriptive statistical analysis of dataset contents

SELECT ?neoplasm ?variation (count (?variation) as ?occurrence)		
WHERE {		
?sample NCIT:Neoplasm_by_Morphology ?neoplasm.		
?somatic_mutation logvd:hasSample ?sample.		
?variation_id rdfs:label ?variation.		
?somatic_mutation logvd:hasVariation ?variation_id.		
}		
GROUP BY ?neoplasm ?variation		
ORDER BY ?neoplasm		
**?neoplasm**	**?variation**	**?occurrence**

Acinar cell carcinoma	NM_000546.1:c.186A>C	1
Acinar cell carcinoma	NM_000546.1:c.408del1	1
Acinar cell carcinoma	NM_000546.1:c.454del1	1
Acinar cell carcinoma	NM_000546.1:c.590T>G	1
Acute leukemia, NOS	NM_000546.1:c.524G>A	2
Acute megakaryoblastic leukemia	NM_000546.1:c.605G>T	1
Acute megakaryoblastic leukemia	NM_000546.1:c.734G>T	1
Acute monocytic leukemia	NM_000546.1:c.584T>C	1
Acute myeloid leukemia with maturation	NM_000546.1:c.743G>A	1
Acute myeloid leukemia with maturation	NM_000546.1:c.862A>T	1
......	......	......

This query selects neoplasm and associated gene variation along with the number of related associations for all somatic mutations in the dataset. The output has been limited to the first 10 results. SPARQL query prefixes are not shown.

**Table 4:** SPARQL query example 2: extraction of complementary data from DBpedia

SELECT ?sample ?patient ?country ?capital			
WHERE {			
?sample logvd:hasIndividual ?patient.			
?sample NCIT:Topography "BRAIN".			
?patient NCIT:Country ?country			
SERVICE <http://dbpedia.org/sparql> {?country <http://dbpedia.org/ontology/capital> ?capital}			
}			
**?sample**	**?patient**	**?country**	**?capital**

samples/112	individual/112	<http://dbpedia.org/resource/Japan>	<http://dbpedia.org/resource/Tokyo>
samples/113	individual/113	<http://dbpedia.org/resource/Japan>	<http://dbpedia.org/resource/Tokyo>
samples/115	individual/115	<http://dbpedia.org/resource/Japan>	<http://dbpedia.org/resource/Tokyo>
samples/116	individual/116	<http://dbpedia.org/resource/Japan>	<http://dbpedia.org/resource/Tokyo>
samples/963	individual/963	<http://dbpedia.org/resource/Japan>	<http://dbpedia.org/resource/Tokyo>
samples/964	individual/964	<http://dbpedia.org/resource/Japan>	<http://dbpedia.org/resource/Tokyo>
samples/1026	individual/1025	<http://dbpedia.org/resource/Canada>	<http://dbpedia.org/resource/Ottawa>
samples/1299	individual/1292	<http://dbpedia.org/resource/Spain>	<http://dbpedia.org/resource/Madrid>
samples/1300	individual/1293	<http://dbpedia.org/resource/Spain>	<http://dbpedia.org/resource/Madrid>
samples/1302	individual/1295	<http://dbpedia.org/resource/Spain>	<http://dbpedia.org/resource/Madrid>
samples/1303	individual/1296	<http://dbpedia.org/resource/Spain>	<http://dbpedia.org/resource/Madrid>
samples/1739	individual/1728	<http://dbpedia.org/resource/Germany>	<http://dbpedia.org/resource/Berlin>
............	............	............	............

This query selects countries and capitals from DBpedia for individuals whose samples were used for the detection of somatic mutations. The SERVICE keyword supports the execution of the query among endpoints distributed across the Web. The output has been limited to the first 12 results. SPARQL query prefixes are not shown.

**Table 5:** SPARQL query example 3: retrieving clinical trials of interest

SELECT DISTINCT ?variation_label ?neoplasm ?clinical_trial		
WHERE {		
SERVICE <http://linkedlifedata.com/sparql> {?clinical_trial relontology:hasInclusionCriteria ?umls}.		
?sample logvd:Sub_topography "Middle third of esophagus".		
?sample NCIT:Neoplasm_by_Morphology ?neoplasm.		
?sample logvd:hasUMLS_neoplasm ?umls.		
?somatic_mutation logvd:hasSample ?sample.		
?variation_id rdfs:label ?variation_label.		
?somatic_mutation logvd:hasVariation ?variation_id.		
}		
ORDER BY ?variation_label ?neoplasm		
**?variation_label**	**?neoplasm**	**?clinical_trial**

NM_000546.1:c.507G > A	Adenocarcinoma, NOS	<http://data.linkedct.org/resource/trials/NCT00001332>
NM_000546.1:c.838A>G	Adenocarcinoma, NOS	<http://data.linkedct.org/resource/trials/NCT00001332>
NM_000546.1:c.507G>A	Adenocarcinoma, NOS	<http://data.linkedct.org/resource/trials/NCT00001428>
NM_000546.1:c.838A>G	Adenocarcinoma, NOS	<http://data.linkedct.org/resource/trials/NCT00001428>
NM_000546.1:c.482C>A	Dysplasia, NOS	<http://data.linkedct.org/resource/trials/NCT00001932>
NM_000546.1:c.482C>A	Dysplasia, NOS	<http://data.linkedct.org/resource/trials/NCT00003076>
NM_000546.1:c.482C>A	Dysplasia, NOS	<http://data.linkedct.org/resource/trials/NCT00003094>
NM_000546.1:c.482C>A	Dysplasia, NOS	<http://data.linkedct.org/resource/trials/NCT00003223>
NM_000546.1:c.482C>A	Dysplasia, NOS	<http://data.linkedct.org/resource/trials/NCT00003593>
NM_000546.1:c.469G>T	Hyperplasia, NOS	<http://data.linkedct.org/resource/trials/NCT00001378>
NM_000546.1:c.469G>T	Hyperplasia, NOS	<http://data.linkedct.org/resource/trials/NCT00001756>
NM_000546.1:c.469G>T	Hyperplasia, NOS	<http://data.linkedct.org/resource/trials/NCT00001760>
NM_000546.1:c.469G>T	Hyperplasia, NOS	<http://data.linkedct.org/resource/trials/NCT00003641>
NM_000546.1:c.422G>A	Squamous cell carcinoma, NOS	<http://data.linkedct.org/resource/trials/NCT00000692>
NM_000546.1:c.451C>G	Squamous cell carcinoma, NOS	<http://data.linkedct.org/resource/trials/NCT00000692>
NM_000546.1:c.469G>T	Squamous cell carcinoma, NOS	<http://data.linkedct.org/resource/trials/NCT00000692>
NM_000546.1:c.474_475ins1	Squamous cell carcinoma, NOS	<http://data.linkedct.org/resource/trials/NCT00001450>
NM_000546.1:c.488A>G	Squamous cell carcinoma, NOS	<http://data.linkedct.org/resource/trials/NCT00001450>

This query selects clinical trials of interest, given a defined sub topography (precise location of the tumor) and shows which variations are involved. The SERVICE keyword supports the execution of the query among endpoints distributed across the Web. The output has been limited to 18 results that were selected with the aim of showing different tumors associated with the given sub topography. SPARQL query prefixes are not shown.

The first query is a simple example involving only the TP53 mutation data. It selects neoplasm-gene variation associations along with the number of their occurrences in the dataset. This query is essentially equivalent to a standard SQL query in the database and shows how similar queries may be performed in order to achieve the same functionalities of a relational database (see Table 3).

The second and third queries show how to perform federated SPARQL queries across distinct datasets. Query federation is expressed by means of the SERVICE keyword in a SPARQL query. This keyword supports the execution of the query on distributed SPARQL endpoints: it causes a sub-pattern of the query to be sent to a named endpoint, instead of being matched on the local dataset.

The second query demonstrates how to retrieve complementary data from DBpedia. Capital towns of countries included in our dataset are retrieved for individuals whose samples were used for the detection of somatic mutations which are present in the mutation dataset (see Table 4).

The last example query shows how to select information from the Linked Clinical Trials (LinkedCT) dataset which is available via the LinkedLifeData endpoint. Given a defined sub topography (precise anatomical location of the origin of the sample) that is put in relation with clinical trials of interest through the shared adoption of the corresponding ULMS code for inclusion criteria in the trial, information on gene variation, neoplasm and clinical trial ID is shown (see Table 5).

### Some statistics on contents of the triple store

Table 6 reports some statistics on contents of our triple store. The number of entities is given by the sum of somatic mutations, gene variations, samples, individuals, and bibliographic references included in the database.

**Table 6:** Main statistics of triple store contents

Triple store size		
Number of entities		85,785
Number of triples		1,002,597

**Number of external URIs**		

LinkedLifeData		25,094
Bio2RDF		2,244
DBpedia		23,015

	Total	50,353

**Number of links to external web pages**		

	Total	2,436

**Shared properties from re-used ontologies**		

**Ontology**	**No. of shared properties**	**Involved triples**

rdf:	1	85,893
rdfs:	3	88,249
owl:	1	2,241
diseasome:	2	2
mio:	2	9,399
bibo:	6	11,385
bibtex:	2	4,478
NCIT:	12	146,553

Total	29	348,200

Statistics of the RDF triple store showing number of triples, entities, external Linked Data URIs, links to external web sites and shared properties from re-used ontologies.

External URIs that specify either Linked Life Data, Bio2RDF or DBpedia entities are included in about 5% of triples. The number of triples including a shared property, i.e. a property that is defined within a re-used ontology, are about one third of the total. It may be noteworthy that there are 9,399 triples including one property from MIO and 146,553 triples with one property from NCIT.

### Availability

Presently, we offer access to our dataset via two distinct modalities. The first interface is a SPARQL endpoint available at: http://bioinformatics.istge.it/logvdsparql/sparql. It is implemented via Joseki and TDB. Interfaces to validators for SPARQL queries and for RDF data are also available. The second interface is a Linked Data representation available at [51]. It is based on the Pubby frontend.

## Discussion

Thanks to some tools that were recently developed, an RDF representation of contents of a relational database can now be easily provided. However, having an RDF representation of a data set of gene mutations is not enough to achieve the desirable integration with other data sets. The main value, and difficulty, lies on the identification of a shared, semantically meaningful, ontology-based representation of variation information.

In our mapping, we have expressed mutations as a central entity which connects sequence variations to individuals. Such mapping adopts, whenever possible, consolidated vocabularies for the description of properties (e.g.: age of patients) or types (e.g.: mutation codes). We have also adopted shared URIs when easily identifiable (e.g. Bio2RDF URIs for Entrez, HGNC, OMIM, etc...).

The resulting RDF has been exposed both via RDF and as Linked Data, hence making the system be a part of the growing Web of Data and a potential bridge between molecular and clinical information.

There are still problems in the development of an effective Linked Data solution for mutation data, in particular the need for URIs which univocally identify mutation (or in alternative a computable definition of equivalent URIs). Current nomenclatures can be the basis for RefSeq version specific URIs, which could be complemented by services to identify equivalent mutations.

At the moment, our model is "application oriented", in the sense that it only reflects the schema and contents of the IARC TP53 Mutation database. But this is also, to our knowledge, the first case of a mutation database expressed in the LOD. For this reason it is, at the same time, a first model from which the community of LSDB and mutation database managers can start developing a general ontology model for gene variation and, hopefully, a recognized reference for future efforts in this direction.

In the future, a remapping of our model in the light of the new developments is then foreseen. A revised version of our prototype, including further shared concepts and all data sets provided by the IARC TP53 Database, is already being developed and we also plan to add more variation databases.

More pertinent or exhaustive ontologies related to variation concepts have to be developed in a community effort. At the same time, standardization of nomenclatures and identifiers must be included, when available.

A clear proof of concept of the advancement that a Linked Data representation of mutation data can currently provide with reference to alternative solutions is hard to assess. There still are some issues with the assessment of the value of publishing resources on the Semantic Web or Linked Data.

The advantages that are offered in terms of easiness and accuracy of data integration can be measured only if a cost component is taken into account. Without this, equivalent solutions can be realized with alternative technologies. It is difficult to measure these aspects, other than in very general qualitative terms.

The Semantic Web is still a "fishing expedition", to cite a definition that was commonly used for functional genomics in its early days [52]. As an intrinsically enabling technology, the Semantic Web platform is built to enable new solutions, rather than addressing some specific and measurable use cases.

In this paper we are proposing to adopt this technology to make mutation data available as Linked Data. This technology will unleash its full potential when a sufficient amount of information will be available.

With our work, we are providing the Web of Data with a class of information which has a pivotal role in enabling translational research. In the short term, the exploitation of SPARQL queries on mutation data sets and other biological databases may support some interesting and useful data retrieval presently not possible. In a longer time frame, reasoning on integrated variation data may also support discoveries towards personalized medicine.

To this purpose, however, much work is still needed, due to the relative isolation of variation data sources and lack of standardization both in terminologies and data schema.

## List of abbreviations used

API: Application Programming Interface; D2RQ: Database to RDF Query; GO: Gene Ontology; HGVS: Human Genome Variation Society; HVP: Human Variome Project; IARC: International Agency for Research on Cancer; LLD: Linked Life Data; LOD: Linked Open Data; LOVD: Leiden Open Variation Database; LSDB: Locus Specific Data Base; MIO: Mutation Impact Ontology; NCI: National Cancer Institute; NCIT: NCI Thesaurus; OMIM: Online Mendelian Inheritance in Man; RDB: Relational Database; RDF: Resource Description Framework; SADI: Semantic Automated Discovery and Integration; SO: Sequence Ontology; SNP: Single Nucleotide Polymorphism; SPARQL: SPARQL Protocol and RDF Query Language; URI: Uniform Resource Identifier; VariO: Variation Ontology.

## Competing interests

The authors declare that they have no competing interests.

## Authors' contributions

PR conceived the work, participated in its design and implementation, and drafted the manuscript. AZ participated in the design of the work, implemented the prototype, and contributed in drafting the manuscript. AS participated in the design of the work, supervised the implementation of the prototype and contributed in drafting the manuscript. All authors read and approved the final manuscript.

## Supplementary Material

Additional file 1 **D2RQ mappings**. Complete list of D2RQ mappings exposing IARC TP53 Mutation database to RDF. The mapping consists in a set of triples which are presented in N3 format. Click here for file
