# Supplementary material for: Towards linked open gene mutations data
Source: BMC Bioinformatics. 2012 Mar 28;13(Suppl 4):S7. doi: 10.1186/1471-2105-13-S4-S7 (PMC3303732; doi:10.1186/1471-2105-13-S4-S7)
Supplement: Additional file 1 — D2RQ mappings. Complete list of D2RQ mappings exposing IARC TP53 Mutation database to RDF. The mapping consists in a set of triples which are presented in N3 format. [file 1471-2105-13-S4-S7-S1.pdf]

```
@prefix map: <http://ml370.istge.it:7777/resource/tp53_vars#> .
@prefix db: <http://ml370.istge.it:7777/resource/> .
@prefix logvd: <http://ml370.istge.it:7777/resource/logvd/> .
@prefix rdf: <http://www.w3.org/1999/02/22-rdf-syntax-ns#> .
@prefix rdfs: <http://www.w3.org/2000/01/rdf-schema#> .
@prefix xsd: <http://www.w3.org/2001/XMLSchema#> .
@prefix d2rq: <http://www.wiwiss.fu-berlin.de/suhl/bizer/D2RQ/0.1#> .
@prefix jdbc: <http://d2rq.org/terms/jdbc/> .
@prefix ncbi_pubmed: <http://www.ncbi.nlm.nih.gov/pubmed/> .
@prefix dbpedia: <http://dbpedia.org/resource/> .
@prefix dbpedia-owl: <http://dbpedia.org/ontology/> .
@prefix dbpediaCategory: <http://dbpedia.org/resource/Category:> .
@prefix bibo: <http://purl.org/ontology/bibo/> .
@prefix bibtex: <http://data.bibbase.org/ontology/#> .
@prefix owl: <http://www.w3.org/2002/07/owl#> .
@prefix NCIT: <http://ncicb.nci.nih.gov/xml/owl/EVS/Thesaurus.owl#> .
@prefix omim: <http://bio2rdf.org/omim:> .
@prefix uniprot: <http://bio2rdf.org/uniprot:> .
@prefix pubmed: <http://bio2rdf.org/pubmed:> .
@prefix hgnc: <http://bio2rdf.org/hgnc:> .
@prefix geneid: <http://bio2rdf.org/geneid:> .
@prefix mio: <http://unbsj.biordf.net/ontologies/mutation-impact-ontology.owl#> .
@prefix diseasome: <http://www4.wiwiss.fu-berlin.de/diseasome/resource/diseasome/> .
@prefix d2r: <http://sites.wiwiss.fu-berlin.de/suhl/bizer/d2r-server/config.rdf#> .


<> a d2r:Server;
    rdfs:label "TP53/IARC D2R Server, National Cancer Research Institute, Genova, Italy";
        d2r:baseURI <http://ml370.istge.it:7777/>;
    d2r:port 7777;
    d2r:documentMetadata [
        rdfs:comment "TP53/IARC D2R Server, National Cancer Research Institute, Genova, Italy";
    ];
        d2r:vocabularyIncludeInstances true;
        d2r:autoReloadMapping true;
    .

map:database a d2rq:Database;
        d2rq:jdbcDriver "com.mysql.jdbc.Driver";
        d2rq:jdbcDSN "jdbc:mysql://localhost/tp53_muts";
        jdbc:autoReconnect "true";
        jdbc:zeroDateTimeBehavior "convertToNull";
#jdbc:keepAlive "3600";                              # sends noop-query every 3600 seconds
#jdbc:keepAliveQuery "SELECT 1";             # optional custom noop-query
        .




# Table mutations15
map:variation a d2rq:ClassMap;
        d2rq:dataStorage map:database;
        d2rq:uriPattern "variation/@@mutations15.Mut_ID@@";
        d2rq:class logvd:variation;
        d2rq:classDefinitionLabel "gene variations";
        d2rq:classDefinitionComment " This is the Gene Variations data set of the IARC TP53
Mutation Database (Rel. 15) "@en;
        .
map:variation__label a d2rq:PropertyBridge;
        d2rq:belongsToClassMap map:variation;
        d2rq:property rdfs:label;
        d2rq:pattern "NM_000546.1:@@mutations15.c_description@@";
        .


map:variation_ExonIntron a d2rq:PropertyBridge;
        d2rq:belongsToClassMap map:variation;
        d2rq:property logvd:ExonIntron;
        d2rq:propertyDefinitionLabel "variations ExonIntron";
        d2rq:propertyDefinitionComment " Location of the mutation in the introns or exons in
TP53 gene. Terms occurring in this column are 1-intron to 11-intron and 2-exon to 11-exon. An i
or e in front means that the mutation is located within the indicated intron or exon with no
information on the precise location.  "@en;
        d2rq:column "mutations15.ExonIntron";
                d2rq:condition "mutations15.ExonIntron != ('NA')";
        d2rq:condition "mutations15.ExonIntron != ('')";
        d2rq:condition "mutations15.ExonIntron != ('-')";
        .
```

```
map:variation_Genomic_nt a d2rq:PropertyBridge;
	d2rq:belongsToClassMap map:variation;
	d2rq:property logvd:Genomic_nt;
	d2rq:propertyDefinitionLabel "variations Genomic_nt";
	d2rq:propertyDefinitionComment " Genomic nucleotide position of the mutation based on
the Genbank NC_000017 (7512445..7531642) reference sequence. "@en;
	d2rq:column "mutations15.Genomic_nt";
		d2rq:condition "mutations15.Genomic_nt != ('NA')";
	d2rq:condition "mutations15.Genomic_nt != ('')";
	d2rq:condition "mutations15.Genomic_nt != ('-')";
	.
map:variation_Codon_number a d2rq:PropertyBridge;
	d2rq:belongsToClassMap map:variation;
	d2rq:property logvd:Codon_number;
	d2rq:propertyDefinitionLabel "variations Codon_number";
	d2rq:propertyDefinitionComment " For mutations in exons, codon number at which the
mutation is located (1-393). If a mutation spans more than one codon, (e.g. tandem mutation or
deletion of several bases) only the first (5') codon is entered. For mutations in introns, 0 is
entered. "@en;
	d2rq:column "mutations15.Codon_number";
		d2rq:condition "mutations15.Codon_number != ('NA')";
	d2rq:condition "mutations15.Codon_number != ('')";
	d2rq:condition "mutations15.Codon_number != ('-')";
	.
map:variation_Splice_site a d2rq:PropertyBridge;
	d2rq:belongsToClassMap map:variation;
	d2rq:property logvd:Splice_site;
	d2rq:propertyDefinitionLabel "variations Splice_site";
	d2rq:propertyDefinitionComment " Annotation on the position of the mutation within
conserved nucleotides of p53 consensus, criptic or alternative splice sites.. "@en;
	d2rq:column "mutations15.Splice_site";
		d2rq:condition "mutations15.Splice_site != ('NA')";
	d2rq:condition "mutations15.Splice_site != ('')";
	d2rq:condition "mutations15.Splice_site != ('-')";
	.
map:variation_CpG_site a d2rq:PropertyBridge;
	d2rq:belongsToClassMap map:variation;
	d2rq:property logvd:CpG_site;
	d2rq:propertyDefinitionLabel "variations CpG_site";
	d2rq:propertyDefinitionComment " Yes or No indicate if the position of the mutation
falls within a CpG sequence or not. "@en;
	d2rq:column "mutations15.CpG_site";
		d2rq:condition "mutations15.CpG_site != ('NA')";
	d2rq:condition "mutations15.CpG_site != ('')";
	d2rq:condition "mutations15.CpG_site != ('-')";
	.
map:variation_Wildtype_Base a d2rq:PropertyBridge;
	d2rq:belongsToClassMap map:variation;
	d2rq:property logvd:Wildtype_Base;
	d2rq:propertyDefinitionLabel "variations Base";
	d2rq:propertyDefinitionComment " Wild-type base at the position of the mutation. "@en;
	d2rq:column "mutations15.Base";
		d2rq:condition "mutations15.Base != ('NA')";
	d2rq:condition "mutations15.Base != ('')";
	d2rq:condition "mutations15.Base != ('-')";
	.
map:variation_Mutant_nucleotide a d2rq:PropertyBridge;
	d2rq:belongsToClassMap map:variation;
	d2rq:property logvd:hasMutant_nucleotide;
	d2rq:propertyDefinitionLabel "variations Mutant_nucleotide";
	d2rq:propertyDefinitionComment " Mutant base. "@en;
	d2rq:column "mutations15.Mutant_nucleotide";
		d2rq:condition "mutations15.Mutant_nucleotide != ('NA')";
	d2rq:condition "mutations15.Mutant_nucleotide != ('')";
	d2rq:condition "mutations15.Mutant_nucleotide != ('-')";
	.
map:variation_Variation_Description a d2rq:PropertyBridge;
	d2rq:belongsToClassMap map:variation;
	d2rq:property logvd:Variation_Description;
	d2rq:propertyDefinitionLabel "variations Description";
	d2rq:propertyDefinitionComment " For substitutions, nucleotide change read from the
coding strand by convention. For deletions and insertions, the number of bases deleted (del) or
inserted (ins) is given. For more complex mutation events, a full description is given as
indicated in the original publication. "@en;
	d2rq:column "mutations15.Description";
		d2rq:condition "mutations15.Description != ('NA')";
	d2rq:condition "mutations15.Description != ('')";
	d2rq:condition "mutations15.Description != ('-')";
```

```
        .
map:variation_c_description a d2rq:PropertyBridge;
        d2rq:belongsToClassMap map:variation;
        d2rq:property logvd:c_description;
        d2rq:propertyDefinitionLabel "variations c_description";
        d2rq:propertyDefinitionComment " Mutation nomenclature according to HGVS standards with
the coding sequence as reference. "@en;
        d2rq:column "mutations15.c_description";
        .
map:variation_Variation_Type a d2rq:PropertyBridge;
        d2rq:belongsToClassMap map:variation;
        d2rq:property logvd:Variation_Type;
        d2rq:propertyDefinitionLabel "variations Type";
        d2rq:propertyDefinitionComment " Nature of the mutation. The terms occurring in this
column are A:T>C:G (A to C or T to G base change), A:T>G:C (A to G or T to C base change),
A:T>T:A (A to T or T to A base change), G:C>A:T (G to A or C to T base change at non CpG sites),
G:C>A:T at CpG (G to A or C to T base change at CpG sites), G:C>C:G (G to C or C to G base
change), G:C>T:A (G to T or C to A base change), tandem (two consecutive base changes), ins
(insertion), del (deletion) and complex (complex changes). "@en;
        d2rq:column "mutations15.Type";
                d2rq:condition "mutations15.Type != ('NA')";
        d2rq:condition "mutations15.Type != ('')";
        d2rq:condition "mutations15.Type != ('-')";
        .
map:variation_hasMut_rate a d2rq:PropertyBridge;
        d2rq:belongsToClassMap map:variation;
        d2rq:property logvd:hasMut_rate;
        d2rq:propertyDefinitionLabel "variations Mutation rate";
        d2rq:propertyDefinitionComment " Substitution rates were calculated for all single base
substitutions in the coding sequence of p53 according to the dinucleotide substitution rates
derived from human-mouse aligned sequences of chromosomes 21 and 10. "@en;
        d2rq:column "mutations15.Mut_rate";
                d2rq:condition "mutations15.Mut_rate != ('NA')";
        d2rq:condition "mutations15.Mut_rate != ('')";
        d2rq:condition "mutations15.Mut_rate != ('-')";
        .
map:variation_WildType_codon a d2rq:PropertyBridge;
        d2rq:belongsToClassMap map:variation;
        d2rq:property logvd:WildType_codon;
        d2rq:propertyDefinitionLabel "variations Wild Type codon";
        d2rq:propertyDefinitionComment " For mutations in exons, normal base sequence of the
codon in which the mutation occurred. "@en;
        d2rq:column "mutations15.WT_codon";
                d2rq:condition "mutations15.WT_codon != ('NA')";
        d2rq:condition "mutations15.WT_codon != ('')";
        d2rq:condition "mutations15.WT_codon != ('-')";
        .
map:variation_hasMutant_codon a d2rq:PropertyBridge;
        d2rq:belongsToClassMap map:variation;
        d2rq:property logvd:hasMutant_codon;
        d2rq:propertyDefinitionLabel "variations Mutant codon";
        d2rq:propertyDefinitionComment " Base sequence of the mutated codon. "@en;
        d2rq:column "mutations15.Mutant_codon";
                d2rq:condition "mutations15.Mutant_codon != ('NA')";
        d2rq:condition "mutations15.Mutant_codon != ('')";
        d2rq:condition "mutations15.Mutant_codon != ('-')";
        .
map:variation_hasWildTypeResidue a d2rq:PropertyBridge;
        d2rq:belongsToClassMap map:variation;
        d2rq:property mio:hasWildTypeResidue;
        d2rq:propertyDefinitionLabel "variations Wild Type AA";
        d2rq:propertyDefinitionComment " Wild Type amino acid encoded at the codon in which the
mutation occurred (three-letter or one-letter amino acid abbreviation). "@en;
        d2rq:column "mutations15.WT_AA";
                d2rq:condition "mutations15.WT_AA != ('NA')";
        d2rq:condition "mutations15.WT_AA != ('')";
        d2rq:condition "mutations15.WT_AA != ('-')";
        .
map:variation_hasMutantResidue a d2rq:PropertyBridge;
        d2rq:belongsToClassMap map:variation;
        d2rq:property mio:hasMutantResidue;
        d2rq:propertyDefinitionLabel "variations Mutant AA";
        d2rq:propertyDefinitionComment " Mutated amino acid encoded at the codon in which the
mutation occurred (three-letter or one-letter amino acid abbreviation). The chain terminating
mutations due to single base substitutions are designated by -stop-. "@en;
        d2rq:column "mutations15.Mutant_AA";
                d2rq:condition "mutations15.Mutant_AA != ('NA')";
        d2rq:condition "mutations15.Mutant_AA != ('')";
```

```
        d2rq:condition "mutations15.Mutant_AA != ('-')";
        .
map:variation_p_description a d2rq:PropertyBridge;
        d2rq:belongsToClassMap map:variation;
        d2rq:property logvd:p_description;
        d2rq:propertyDefinitionLabel "variations Protein Mutation Description";
        d2rq:propertyDefinitionComment " Mutation description at the protein level as
recommended by HGVS. "@en;
        d2rq:column "mutations15.ProtDescription";
                d2rq:condition "mutations15.ProtDescription != ('-')";
        d2rq:condition "mutations15.ProtDescription != ('NA')";
        d2rq:condition "mutations15.ProtDescription != ('p.?')";
        .
map:variation_hasMut_rateAA a d2rq:PropertyBridge;
        d2rq:belongsToClassMap map:variation;
        d2rq:property logvd:hasMut_rate_AA;
        d2rq:propertyDefinitionLabel "variations Mutation rate of AA";
        d2rq:propertyDefinitionComment " Mutation rate of amino-acid substitution calculated by
summing up the nucleotide substitution rates. This value is only valid for amino-acid
substitutions resulting from single nucleotide substitutions. "@en;
        d2rq:column "mutations15.Mut_rateAA";
                d2rq:condition "mutations15.Mut_rateAA != ('NA')";
        d2rq:condition "mutations15.Mut_rateAA != ('')";
        d2rq:condition "mutations15.Mut_rateAA != ('-')";
        .
map:variation_hasEffect a d2rq:PropertyBridge;
        d2rq:belongsToClassMap map:variation;
        d2rq:property logvd:hasEffect;
        d2rq:propertyDefinitionLabel "variations Effect";
        d2rq:propertyDefinitionComment " Effect of the mutation. The terms occurring in this
column are: missense (change of one amino-acid), nonsense (stop codon), FS (frameshift), silent
(no change in the protein sequence), splice (mutations located in the two first and two last
conserved nucleotides of the introns and are thus predicted to alter splicing, or mutations that
have been shown to alter splicing experimentally), other (inframe deletions or insertions,
mutations in introns). "@en;
        d2rq:column "mutations15.Effect";
                d2rq:condition "mutations15.Effect != ('NA')";
        d2rq:condition "mutations15.Effect != ('')";
        d2rq:condition "mutations15.Effect != ('-')";
        .
map:variation_Polymorphism a d2rq:PropertyBridge;
        d2rq:belongsToClassMap map:variation;
        d2rq:property logvd:Polymorphism;
        d2rq:propertyDefinitionLabel "variations Polymorphism";
        d2rq:propertyDefinitionComment " Polymorphic status of the gene variation. "@en;
        d2rq:column "mutations15.Polymorphism";
                d2rq:condition "mutations15.Polymorphism != ('NA')";
        d2rq:condition "mutations15.Polymorphism != ('')";
        d2rq:condition "mutations15.Polymorphism != ('-')";
        .
map:variation_Validation_type a d2rq:PropertyBridge;
        d2rq:belongsToClassMap map:variation;
        d2rq:property logvd:Validation_type;
        d2rq:propertyDefinitionLabel "variations Validation";
        d2rq:propertyDefinitionComment " Type of validation for polymorphism, as indicated in
NCBI SNP database. "@en;
        d2rq:column "mutations15.Validation";
                d2rq:condition "mutations15.Validation != ('NA')";
        d2rq:condition "mutations15.Validation != ('')";
        d2rq:condition "mutations15.Validation != ('-')";
        .
map:variation_dbSNP_link a d2rq:PropertyBridge;
        d2rq:belongsToClassMap map:variation;
        d2rq:property logvd:dbSNP_link;
        d2rq:propertyDefinitionLabel "variations dbSNP link";
        d2rq:propertyDefinitionComment " Link to NCBI SNP database. "@en;
        d2rq:uriPattern
"http://www.ncbi.nlm.nih.gov/projects/SNP/snp_ref.cgi?rs=@@mutations15.SNPlink@@";
         d2rq:condition "mutations15.SNPlink != ('NA')";
        d2rq:condition "mutations15.SNPlink != ('')";
        d2rq:condition "mutations15.SNPlink != ('-')";
        .
map:variation_Residue_function a d2rq:PropertyBridge;
        d2rq:belongsToClassMap map:variation;
        d2rq:property logvd:Residue_function;
        d2rq:propertyDefinitionLabel "variations Residue function";
```

```
        d2rq:propertyDefinitionComment " Known function of the wild-type residue. When the
function is not known but the structure is known, the solvent accessibility (SA) of the residue
is indicated by the terms buried, exposed or partially exposed. "@en;
        d2rq:column "mutations15.Residue_function";
                d2rq:condition "mutations15.Residue_function != ('NA')";
        d2rq:condition "mutations15.Residue_function != ('')";
        d2rq:condition "mutations15.Residue_function != ('-')";
        .
map:variation_Domain_function a d2rq:PropertyBridge;
        d2rq:belongsToClassMap map:variation;
        d2rq:property logvd:Domain_function;
        d2rq:propertyDefinitionLabel "variations Domain function";
        d2rq:propertyDefinitionComment " Function of the domain in which the mutated residue is
located. "@en;
        d2rq:column "mutations15.Domain_function";
                d2rq:condition "mutations15.Domain_function != ('NA')";
        d2rq:condition "mutations15.Domain_function != ('')";
        d2rq:condition "mutations15.Domain_function != ('-')";
        .
map:variation_Structural_motif a d2rq:PropertyBridge;
        d2rq:belongsToClassMap map:variation;
        d2rq:property logvd:Variation_motif;
        d2rq:propertyDefinitionLabel "variations Structural motif";
        d2rq:propertyDefinitionComment " 2D and 3D motifs where the mutation is located
according to structures described in Cho et al. (1994) and May and May (1999). "@en;
        d2rq:column "mutations15.Structural_motif";
                d2rq:condition "mutations15.Structural_motif != ('NA')";
        d2rq:condition "mutations15.Structural_motif != ('')";
        d2rq:condition "mutations15.Structural_motif != ('-')";
        .
map:variation_Grantham_variation a d2rq:PropertyBridge;
        d2rq:belongsToClassMap map:variation;
        d2rq:property logvd:Grantham_variation;
        d2rq:propertyDefinitionLabel "variations Grantham variation";
        d2rq:propertyDefinitionComment " Grantham variation. GV is a measure of the amount of
observed biochemical variation at a particular position in a multiple sequence alignment. GV was
calculated with an alignment containing 9 sequences of p53 from fish to placental mammals with
the A-GVGD program. "@en;
        d2rq:column "mutations15.GV";
                d2rq:condition "mutations15.GV != ('NA')";
        d2rq:condition "mutations15.GV != ('')";
        d2rq:condition "mutations15.GV != ('-')";
        .
map:variation_Grantham_deviation a d2rq:PropertyBridge;
        d2rq:belongsToClassMap map:variation;
        d2rq:property logvd:Grantham_deviation;
        d2rq:propertyDefinitionLabel "variations Grantham deviation";
        d2rq:propertyDefinitionComment " Grantham deviation. GD is a measure of the deviation of
the mutated residue from the different types of residues observed at that postion in a multiple
sequence alignment. It is derived from GV and the Grantham matrix. "@en;
        d2rq:column "mutations15.GD";
                d2rq:condition "mutations15.GD != ('NA')";
        d2rq:condition "mutations15.GD != ('')";
        d2rq:condition "mutations15.GD != ('-')";
        .
map:variation_AGVGDClass a d2rq:PropertyBridge;
        d2rq:belongsToClassMap map:variation;
        d2rq:property logvd:AGVGDClass;
        d2rq:propertyDefinitionLabel "variations AGVGD Class";
        d2rq:propertyDefinitionComment " Prediction of functional impact based on protein
sequence conservation, taking into acount GV and GD. Mutations are classified as -neutral-, -
deleterious- or -unclassified-. "@en;
        d2rq:column "mutations15.AGVGDClass";
                d2rq:condition "mutations15.AGVGDClass != ('NA')";
        d2rq:condition "mutations15.AGVGDClass != ('')";
        d2rq:condition "mutations15.AGVGDClass != ('-')";
        .
map:variation_SIFTClass a d2rq:PropertyBridge;
        d2rq:belongsToClassMap map:variation;
        d2rq:property logvd:SIFTClass;
        d2rq:propertyDefinitionLabel "variations SIFT Class";
        d2rq:propertyDefinitionComment " Functional classification based on SIFT program using
the same sequence alignment as for AGVGDclass and program default settings. Missense mutations
are classified as -deleterious- or -neutral-. "@en;
        d2rq:column "mutations15.SIFTClass";
                d2rq:condition "mutations15.SIFTClass != ('NA')";
        d2rq:condition "mutations15.SIFTClass != ('')";
        d2rq:condition "mutations15.SIFTClass != ('-')";
```

```
        .
map:variation_WAF1nWT a d2rq:PropertyBridge;
        d2rq:belongsToClassMap map:variation;
        d2rq:property logvd:WAF1nWT;
        d2rq:propertyDefinitionLabel "variations WAF1nWT";
        d2rq:propertyDefinitionComment " p21-WAF1 promoter-specific transcriptional activity
measured in yeast functional assays and expressed as percent of wild-type activity.  "@en;
        d2rq:column "mutations15.WAF1nWT";
                d2rq:condition "mutations15.WAF1nWT != ('NA')";
        d2rq:condition "mutations15.WAF1nWT != ('')";
        d2rq:condition "mutations15.WAF1nWT != ('-')";
        .
map:variation_MDM2nWT a d2rq:PropertyBridge;
        d2rq:belongsToClassMap map:variation;
        d2rq:property logvd:MDM2nWT;
        d2rq:propertyDefinitionLabel "variations MDM2nWT";
        d2rq:propertyDefinitionComment " MDM2 promoter-specific transcriptional activity
measured in yeast functional assays and expressed as percent of wild-type activity. "@en;
        d2rq:column "mutations15.MDM2nWT";
                d2rq:condition "mutations15.MDM2nWT != ('NA')";
        d2rq:condition "mutations15.MDM2nWT != ('')";
        d2rq:condition "mutations15.MDM2nWT != ('-')";
        .
map:variation_BAXnWT a d2rq:PropertyBridge;
        d2rq:belongsToClassMap map:variation;
        d2rq:property logvd:BAXnWT;
        d2rq:propertyDefinitionLabel "variations BAXnWT";
        d2rq:propertyDefinitionComment " BAX promoter-specific transcriptional activity measured
in yeast functional assays and expressed as percent of wild-type activity.  "@en;
        d2rq:column "mutations15.BAXnWT";
                d2rq:condition "mutations15.BAXnWT != ('NA')";
        d2rq:condition "mutations15.BAXnWT != ('')";
        d2rq:condition "mutations15.BAXnWT != ('-')";
        .
map:variation_h1433snWT a d2rq:PropertyBridge;
        d2rq:belongsToClassMap map:variation;
        d2rq:property logvd:h1433snWT;
        d2rq:propertyDefinitionLabel "variations h1433snWT";
        d2rq:propertyDefinitionComment " 14-3-3sigma promoter-specific transcriptional activity
measured in yeast functional assays and expressed as percent of wild-type activity.  "@en;
        d2rq:column "mutations15.h1433snWT";
                d2rq:condition "mutations15.h1433snWT != ('NA')";
        d2rq:condition "mutations15.h1433snWT != ('')";
        d2rq:condition "mutations15.h1433snWT != ('-')";
        .
map:variation_AIP1nWT a d2rq:PropertyBridge;
        d2rq:belongsToClassMap map:variation;
        d2rq:property logvd:AIP1nWT;
        d2rq:propertyDefinitionLabel "variations AIP1nWT";
        d2rq:propertyDefinitionComment " p53AIP1 promoter-specific transcriptional activity
measured in yeast functional assays and expressed as percent of wild-type activity.  "@en;
        d2rq:column "mutations15.AIP1nWT";
                d2rq:condition "mutations15.AIP1nWT != ('NA')";
        d2rq:condition "mutations15.AIP1nWT != ('')";
        d2rq:condition "mutations15.AIP1nWT != ('-')";
        .
map:variation_GADD45nWT a d2rq:PropertyBridge;
        d2rq:belongsToClassMap map:variation;
        d2rq:property logvd:GADD45nWT;
        d2rq:propertyDefinitionLabel "variations GADD45nWT";
        d2rq:propertyDefinitionComment " GADD45 promoter-specific transcriptional activity
measured in yeast functional assays and expressed as percent of wild-type activity.  "@en;
        d2rq:column "mutations15.GADD45nWT";
                d2rq:condition "mutations15.GADD45nWT != ('NA')";
        d2rq:condition "mutations15.GADD45nWT != ('')";
        d2rq:condition "mutations15.GADD45nWT != ('-')";
        .
map:variation_NOXAnWT a d2rq:PropertyBridge;
        d2rq:belongsToClassMap map:variation;
        d2rq:property logvd:NOXAnWT;
        d2rq:propertyDefinitionLabel "variations NOXAnWT";
        d2rq:propertyDefinitionComment " Noxa promoter-specific transcriptional activity
measured in yeast functional assays and expressed as percent of wild-type activity."@en;
        d2rq:column "mutations15.NOXAnWT";
                d2rq:condition "mutations15.NOXAnWT != ('NA')";
        d2rq:condition "mutations15.NOXAnWT != ('')";
        d2rq:condition "mutations15.NOXAnWT != ('-')";
        .
```

```
map:variation_P53R2nWT a d2rq:PropertyBridge;
	d2rq:belongsToClassMap map:variation;
	d2rq:property logvd:P53R2nWT;
	d2rq:propertyDefinitionLabel "variations P53R2nWT";
	d2rq:propertyDefinitionComment " P53R2 promoter-specific transcriptional activity
measured in yeast functional assays and expressed as percent of wild-type activity."@en;
	d2rq:column "mutations15.P53R2nWT";
		d2rq:condition "mutations15.P53R2nWT != ('NA')";
	d2rq:condition "mutations15.P53R2nWT != ('')";
	d2rq:condition "mutations15.P53R2nWT != ('-')";
	.
map:variation_TransactivationClass a d2rq:PropertyBridge;
	d2rq:belongsToClassMap map:variation;
	d2rq:property logvd:TransactivationClass;
	d2rq:propertyDefinitionLabel "variations TransactivationClass";
	d2rq:propertyDefinitionComment " Functional classification based on the overall
transcriptional activity on 8 different promoters as measured by Kato et al."@en;
	d2rq:column "mutations15.TransactivationClass";
		d2rq:condition "mutations15.TransactivationClass != ('NA')";
	d2rq:condition "mutations15.TransactivationClass != ('')";
	d2rq:condition "mutations15.TransactivationClass != ('-')";
	.
map:variation_StructureFunctionClass a d2rq:PropertyBridge;
	d2rq:belongsToClassMap map:variation;
	d2rq:property logvd:StructureFunctionClass;
	d2rq:propertyDefinitionLabel "variations StructureFunctionClass";
	d2rq:propertyDefinitionComment " Functional predictions derived from a computer model
that takes into account the 3D structure of WT and mutant proteins and is trained on the
transactivaton dataset from Kato et al. Mutations are classified as -functional- or -non-
functional-. "@en;
	d2rq:column "mutations15.StructureFunctionClass";
		d2rq:condition "mutations15.StructureFunctionClass != ('NA')";
	d2rq:condition "mutations15.StructureFunctionClass != ('')";
	d2rq:condition "mutations15.StructureFunctionClass != ('-')";
	.
map:variation_DNEclass a d2rq:PropertyBridge;
	d2rq:belongsToClassMap map:variation;
	d2rq:property logvd:DNEclass;
	d2rq:propertyDefinitionLabel "variations DNEclass";
	d2rq:propertyDefinitionComment " Dominant-negative activity over wild-type p53. "@en;
	d2rq:column "mutations15.DNEclass";
		d2rq:condition "mutations15.DNEclass != ('NA')";
	d2rq:condition "mutations15.DNEclass != ('')";
	d2rq:condition "mutations15.DNEclass != ('-')";
	.
map:variation_SomaticCount a d2rq:PropertyBridge;
	d2rq:belongsToClassMap map:variation;
	d2rq:property logvd:SomaticCount;
	d2rq:propertyDefinitionLabel "variations SomaticCount";
	d2rq:propertyDefinitionComment " The number of occurences as somatic event for each gene
variation. "@en;
	d2rq:column "mutations15.SomaticCount";
		d2rq:condition "mutations15.SomaticCount != ('NA')";
	d2rq:condition "mutations15.SomaticCount != ('')";
	d2rq:condition "mutations15.SomaticCount != ('-')";
	.

# Table somatic15
map:somatic_mutation a d2rq:ClassMap;
	d2rq:dataStorage map:database;
	d2rq:uriPattern "somatic_mutation/@@somatic15.Mutation_ID@@";
	d2rq:class logvd:somatic_mutation;
	d2rq:classDefinitionLabel "somatic mutations";
	d2rq:classDefinitionComment " Somatic mutations dataset: Type of TP53 somatic mutations
in human cancer.  The dataset of Somatic Mutation contains exclusively TP53 somatic mutations
that have been identified in human tissues by sequencing and published in the peer-reviewed
literature. This includes mutations found in normal, pre-neoplastic and neoplastic tissues,
including metastases, as well as in cell lines derived from such tissues. The database does not
include (1) individual data on human tissues that are reported as negative with respect to p53
mutation, (2) mutations not precisely identified by sequencing (e.g. mutations identified only
by SSCP, DGGE or restriction digestion), (3) experimentally-induced mutations in tumor cells or
cell lines in vitro, (4) p53 mutations in animal tumors. "@en;
	.
map:somatic_mutation__label a d2rq:PropertyBridge;
	d2rq:belongsToClassMap map:somatic_mutation;
	d2rq:property rdfs:label;
	d2rq:pattern "somatic_mutation/@@somatic15.Mutation_ID@@";
	.
```

```
map:somatic_mutation_MUT_ID a d2rq:PropertyBridge;
       d2rq:belongsToClassMap map:somatic_mutation;
       d2rq:property logvd:hasVariation;
       d2rq:propertyDefinitionLabel "somatic MUT_ID";
       d2rq:propertyDefinitionComment " Unique identifier of each gene variation reported in
the database. This identifier is used in all datasets. "@en;
       d2rq:refersToClassMap map:variation;
    d2rq:join "somatic15.MUT_ID => mutations15.MUT_ID";
       .


map:somatic_mutation_c_description a d2rq:PropertyBridge;
       d2rq:belongsToClassMap map:somatic_mutation;
       d2rq:property logvd:c_description;
       d2rq:propertyDefinitionLabel "somatic c_description";
       d2rq:propertyDefinitionComment " Mutation nomenclature according to HGVS standards with
the coding sequence as reference. "@en;
       d2rq:column "somatic15.c_description";
       .


map:somatic_mutation_Structural_motif a d2rq:PropertyBridge;
       d2rq:belongsToClassMap map:somatic_mutation;
       d2rq:property logvd:Structural_motif;
       d2rq:propertyDefinitionLabel "somatic Structural_motif";
       d2rq:propertyDefinitionComment " 2D and 3D motifs where the mutation is located
according to structures described in Cho et al. (1994) and May and May (1999). "@en;
       d2rq:column "somatic15.Structural_motif";
       .
map:somatic_mutation_Putative_stop a d2rq:PropertyBridge;
       d2rq:belongsToClassMap map:somatic_mutation;
       d2rq:property logvd:Putative_stop;
       d2rq:propertyDefinitionLabel "somatic Putative_stop";
       d2rq:propertyDefinitionComment " Putative stop. "@en;
       d2rq:column "somatic15.Putative_stop";
               d2rq:condition "somatic15.Putative_stop != ('NA')";
       d2rq:condition "somatic15.Putative_stop != ('')";
       d2rq:condition "somatic15.Putative_stop != ('-')";
       .

map:somatic_mutation_Sample_ID a d2rq:PropertyBridge;
       d2rq:belongsToClassMap map:somatic_mutation;
       d2rq:property logvd:hasSample;
       d2rq:propertyDefinitionLabel "somatic Sample ref";
       d2rq:propertyDefinitionComment " Unique sample identification number. This number allows
the automatic retrieval of samples with multiple mutations. "@en;
       d2rq:refersToClassMap map:sample;
    d2rq:join "somatic15.Sample_ID => somatic15.Sample_ID";
       .


map:somatic_mutation_Ref_ID a d2rq:PropertyBridge;
       d2rq:belongsToClassMap map:somatic_mutation;
       d2rq:property logvd:hasReference;
       d2rq:propertyDefinitionLabel "somatic Pubblication ref";
       d2rq:propertyDefinitionComment " Unique identification number for a reference. "@en;
       d2rq:refersToClassMap map:somatic_ref;
    d2rq:join "somatic15.Ref_ID => SomaticRef15.Ref_ID";
       .


# Table SomaticRef15
map:somatic_ref a d2rq:ClassMap;
       d2rq:dataStorage map:database;
       d2rq:uriPattern "somatic_ref/@@SomaticRef15.Ref_ID@@";
       d2rq:class logvd:somatic_ref;
       d2rq:classDefinitionLabel "Somatic mutations References";
       d2rq:classDefinitionComment " TP53SomaticRefR15 daataset that includes the references in
which are described the mutations. "@en;
       .
map:somatic_ref__label a d2rq:PropertyBridge;
       d2rq:belongsToClassMap map:somatic_ref;
       d2rq:property rdfs:label;
       d2rq:pattern "somatic_ref/@@SomaticRef15.Ref_ID@@";
       .
```

```
map:somatic_ref_Cross_Ref_ID a d2rq:PropertyBridge;
	d2rq:belongsToClassMap map:somatic_ref;
	d2rq:property logvd:hasCross_Ref_ID;
	d2rq:propertyDefinitionLabel "SomaticRef Cross_Ref_ID";
	d2rq:propertyDefinitionComment "Ref ID of a reference containing related data or
additional information."@en;
	 d2rq:condition "SomaticRef15.Cross_Ref_ID != ('0')";
	d2rq:condition "SomaticRef15.Cross_Ref_ID != ('-')";
	d2rq:refersToClassMap map:somatic_ref;
	d2rq:join "SomaticRef15.Cross_Ref_ID => SomaticRef15.Cross_Ref_ID";
	.

map:somatic_ref_Title a d2rq:PropertyBridge;
	d2rq:belongsToClassMap map:somatic_ref;
	d2rq:property bibtex:hasTitle;
	d2rq:propertyDefinitionLabel "SomaticRef Title";
	d2rq:propertyDefinitionComment "Title of the publication."@en;
	d2rq:column "SomaticRef15.Title";
	.
map:somatic_ref_Authors a d2rq:PropertyBridge;
	d2rq:belongsToClassMap map:somatic_ref;
	d2rq:property bibo:authorList;
	d2rq:propertyDefinitionLabel "List of Authors";
	d2rq:propertyDefinitionComment "List of Authors"@en;
	d2rq:column "SomaticRef15.Authors";
	.
map:somatic_ref_Year a d2rq:PropertyBridge;
	d2rq:belongsToClassMap map:somatic_ref;
	d2rq:property bibtex:hasYear;
	d2rq:propertyDefinitionLabel "SomaticRef Year of publication";
	d2rq:propertyDefinitionComment "Year of publication."@en;
	d2rq:column "SomaticRef15.Year";
	.
map:somatic_ref_Journal a d2rq:PropertyBridge;
	d2rq:belongsToClassMap map:somatic_ref;
	d2rq:property bibo:Journal;
	d2rq:propertyDefinitionLabel "SomaticRef Journal";
	d2rq:propertyDefinitionComment "Name of Journal."@en;
	d2rq:column "SomaticRef15.Journal";
		d2rq:condition "SomaticRef15.Journal != ('NA')";
	d2rq:condition "SomaticRef15.Journal != ('')";
	d2rq:condition "SomaticRef15.Journal != ('-')";
	.
map:somatic_ref_Volume a d2rq:PropertyBridge;
	d2rq:belongsToClassMap map:somatic_ref;
	d2rq:property bibo:volume;
	d2rq:propertyDefinitionLabel "SomaticRef Volume";
	d2rq:propertyDefinitionComment "Volume number."@en;
	d2rq:column "SomaticRef15.Volume";
		d2rq:condition "SomaticRef15.Volume != ('NA')";
	d2rq:condition "SomaticRef15.Volume != ('')";
	d2rq:condition "SomaticRef15.Volume != ('-')";
	.
map:somatic_ref_Start_page a d2rq:PropertyBridge;
	d2rq:belongsToClassMap map:somatic_ref;
	d2rq:property bibo:pageStart;
	d2rq:propertyDefinitionLabel "SomaticRef Start_page";
	d2rq:propertyDefinitionComment "Starting Page."@en;
	d2rq:column "SomaticRef15.Start_page";
		d2rq:condition "SomaticRef15.Start_page != ('NA')";
	d2rq:condition "SomaticRef15.Start_page != ('')";
	d2rq:condition "SomaticRef15.Start_page != ('-')";
	.
map:somatic_ref_End_page a d2rq:PropertyBridge;
	d2rq:belongsToClassMap map:somatic_ref;
	d2rq:property bibo:pageEnd;
	d2rq:propertyDefinitionLabel "SomaticRef End_page";
	d2rq:propertyDefinitionComment "Ending Page."@en;
	d2rq:column "SomaticRef15.End_page";
		d2rq:condition "SomaticRef15.End_page != ('NA')";
	d2rq:condition "SomaticRef15.End_page != ('')";
	d2rq:condition "SomaticRef15.End_page != ('-')";
	.
map:somatic_ref_PubMed a d2rq:PropertyBridge;
	d2rq:belongsToClassMap map:somatic_ref;
	d2rq:property owl:sameAs;
	d2rq:propertyDefinitionLabel "SomaticRef PubMed";
	d2rq:propertyDefinitionComment "PubMed Id ref."@en;
```

```
        d2rq:uriPattern "http://bio2rdf.org/pubmed:@@SomaticRef15.PubMed@@";
        d2rq:additionalClassDefinitionProperty map:somatic_ref_PubMedEquivalence;
        .
map:somatic_ref_PubMedEquivalence a d2rq:AdditionalProperty;
        d2rq:belongsToClassMap map:somatic_ref;
        d2rq:property rdfs:seeAlso;
        d2rq:propertyDefinitionLabel "SomaticRef ncbi PubMed";
        d2rq:propertyDefinitionComment "PubMed Id ref."@en;
        d2rq:uriPattern "http://www.ncbi.nlm.nih.gov/pubmed/@@SomaticRef15.PubMed@@";
        .

map:somatic_ref_Comment a d2rq:PropertyBridge;
        d2rq:belongsToClassMap map:somatic_ref;
        d2rq:property bibo:annotates;
        d2rq:propertyDefinitionLabel "SomaticRef Comment";
        d2rq:propertyDefinitionComment "Any relevant information."@en;
        d2rq:column "SomaticRef15.Comment";
                d2rq:condition "SomaticRef15.Comment != ('NA')";
        d2rq:condition "SomaticRef15.Comment != ('')";
        d2rq:condition "SomaticRef15.Comment != ('-')";
        .

map:somatic_ref_Exclude_analysis a d2rq:PropertyBridge;
        d2rq:belongsToClassMap map:somatic_ref;
        d2rq:property logvd:Exclude_analysis;
        d2rq:propertyDefinitionLabel "SomaticRef Exclude_analysis";
        d2rq:propertyDefinitionComment " Papers that we recommend to exclude from any analysis
because of poor data quality. "@en;
        d2rq:column "SomaticRef15.Exclude_analysis";
                d2rq:condition "SomaticRef15.Exclude_analysis != ('NA')";
        d2rq:condition "SomaticRef15.Exclude_analysis != ('')";
        d2rq:condition "SomaticRef15.Exclude_analysis != ('-')";
        .



# From Table somatic15  to patients demographic details
map:individual a d2rq:ClassMap;
        d2rq:dataStorage map:database;
        d2rq:uriPattern "individual/@@somatic15.Individual_ID@@";
        d2rq:class logvd:individual;
        d2rq:classDefinitionLabel "Demographic information of patient-individual.";
        d2rq:classDefinitionComment "Patients demographic details and description of the patient
origin and life-style. This dataset contains heterogeneous notes, usually comments emphasized by
authors reporting the mutations."@en;
        .
map:individual__label a d2rq:PropertyBridge;
        d2rq:belongsToClassMap map:individual;
        d2rq:property rdfs:label;
        d2rq:pattern "individual/@@somatic15.Individual_ID@@";
        .


map:individual_Sex a d2rq:PropertyBridge;
        d2rq:belongsToClassMap map:individual;
        d2rq:property NCIT:Sex_or_Gender;
        d2rq:propertyDefinitionLabel "individual Sex";
        d2rq:propertyDefinitionComment "Sex of the patient."@en;
        d2rq:column "somatic15.Sex";
                d2rq:condition "somatic15.Sex != ('NA')";
        d2rq:condition "somatic15.Sex != ('')";
        d2rq:condition "somatic15.Sex != ('-')";
        .
map:individual_Age a d2rq:PropertyBridge;
        d2rq:belongsToClassMap map:individual;
        d2rq:property NCIT:Age;
        d2rq:propertyDefinitionLabel "individual Age";
        d2rq:propertyDefinitionComment " "@en;
        d2rq:column "somatic15.Age";
                d2rq:condition "somatic15.Age != ('NA')";
        d2rq:condition "somatic15.Age != ('')";
        d2rq:condition "somatic15.Age != ('-')";
        .
map:individual_Ethnicity a d2rq:PropertyBridge;
        d2rq:belongsToClassMap map:individual;
        d2rq:property NCIT:Ethnicity;
        d2rq:propertyDefinitionLabel "individual Ethnicity";
        d2rq:propertyDefinitionComment " "@en;
```

```
        d2rq:column "somatic15.Ethnicity";
                d2rq:condition "somatic15.Ethnicity != ('NA')";
        d2rq:condition "somatic15.Ethnicity != ('')";
        d2rq:condition "somatic15.Ethnicity != ('-')";
        .
map:individual_Population a d2rq:PropertyBridge;
        d2rq:belongsToClassMap map:individual;
        d2rq:property NCIT:Population_Group;
        d2rq:propertyDefinitionLabel "individual Population";
        d2rq:propertyDefinitionComment " "@en;
        d2rq:column "somatic15.Population";
                d2rq:condition "somatic15.Population != ('NA')";
        d2rq:condition "somatic15.Population != ('')";
        d2rq:condition "somatic15.Population != ('-')";
        .
map:individual_Country a d2rq:PropertyBridge;
        d2rq:belongsToClassMap map:individual;
        d2rq:property NCIT:Country;
        d2rq:propertyDefinitionLabel "individual Country";
        d2rq:propertyDefinitionComment " "@en;
               d2rq:uriPattern "http://dbpedia.org/resource/@@somatic15.Country@@";
        d2rq:condition "somatic15.Country != ('NA')";
        d2rq:condition "somatic15.Country != ('')";
        d2rq:condition "somatic15.Country != ('-')";
        .
map:individual_Geo_area a d2rq:PropertyBridge;
        d2rq:belongsToClassMap map:individual;
        d2rq:property NCIT:Geographic_Area;
        d2rq:propertyDefinitionLabel "individual Geo_area";
        d2rq:propertyDefinitionComment " "@en;
        d2rq:column "somatic15.Geo_area";
                d2rq:condition "somatic15.Geo_area != ('NA')";
        d2rq:condition "somatic15.Geo_area != ('')";
        d2rq:condition "somatic15.Geo_area != ('-')";
        .
map:individual_TP53polymorphism a d2rq:PropertyBridge;
        d2rq:belongsToClassMap map:individual;
        d2rq:property logvd:TP53polymorphism;
        d2rq:propertyDefinitionLabel "somatic TP53polymorphism";
        d2rq:propertyDefinitionComment " Presence of a polymorphism in TP53 gene. "@en;
        d2rq:column "somatic15.TP53polymorphism";
                d2rq:condition "somatic15.TP53polymorphism != ('NA')";
        d2rq:condition "somatic15.TP53polymorphism != ('')";
        d2rq:condition "somatic15.TP53polymorphism != ('-')";
        .
map:individual_germline_mutation a d2rq:PropertyBridge;
        d2rq:belongsToClassMap map:individual;
        d2rq:property logvd:germline_mutation;
        d2rq:propertyDefinitionLabel "somatic germline mutation";
        d2rq:propertyDefinitionComment " Presence of a germline mutation in TP53 or any other
gene. "@en;
        d2rq:column "somatic15.Germline_mutation";
                d2rq:condition "somatic15.Germline_mutation != ('NA')";
        d2rq:condition "somatic15.Germline_mutation != ('')";
        d2rq:condition "somatic15.Germline_mutation != ('-')";
        .
map:individual_Family_history a d2rq:PropertyBridge;
        d2rq:belongsToClassMap map:individual;
        d2rq:property logvd:Family_history;
        d2rq:propertyDefinitionLabel "individual Family_history";
        d2rq:propertyDefinitionComment " Information on the presence or absence of cancers in
the family of the patient. "@en;
        d2rq:column "somatic15.Family_history";
                d2rq:condition "somatic15.Family_history != ('NA')";
        d2rq:condition "somatic15.Family_history != ('')";
        d2rq:condition "somatic15.Family_history != ('-')";
        .
map:individual_Tobacco a d2rq:PropertyBridge;
        d2rq:belongsToClassMap map:individual;
        d2rq:property NCIT:Tobacco_Use;
        d2rq:propertyDefinitionLabel "individual Tobacco";
        d2rq:propertyDefinitionComment " Information on the smoking status of the patient. "@en;
        d2rq:column "somatic15.Tobacco";
                d2rq:condition "somatic15.Tobacco != ('NA')";
        d2rq:condition "somatic15.Tobacco != ('')";
        d2rq:condition "somatic15.Tobacco != ('-')";
        .
map:individual_Alcohol a d2rq:PropertyBridge;
```

```
        d2rq:belongsToClassMap map:individual;
        d2rq:property NCIT:Alcohol_Consumption;
        d2rq:propertyDefinitionLabel "individual Alcohol";
        d2rq:propertyDefinitionComment " Information on the drinking status of the patient.
"@en;
        d2rq:column "somatic15.Alcohol";
                d2rq:condition "somatic15.Alcohol != ('NA')";
        d2rq:condition "somatic15.Alcohol != ('')";
        d2rq:condition "somatic15.Alcohol != ('-')";
        .
map:individual_Exposure a d2rq:PropertyBridge;
        d2rq:belongsToClassMap map:individual;
        d2rq:property logvd:Exposure;
        d2rq:propertyDefinitionLabel "individual Exposure";
        d2rq:propertyDefinitionComment " Risk factors to which the patient has been exposed to,
such as aflatoxins, radon, thorotrast, etc"@en;
        d2rq:column "somatic15.Exposure";
                d2rq:condition "somatic15.Exposure != ('NA')";
        d2rq:condition "somatic15.Exposure != ('')";
        d2rq:condition "somatic15.Exposure != ('-')";
        .
map:individual_Infectious_agent a d2rq:PropertyBridge;
        d2rq:belongsToClassMap map:individual;
        d2rq:property logvd:Infectious_agent;
        d2rq:propertyDefinitionLabel "individual Infectious_agent";
        d2rq:propertyDefinitionComment " Pathogen (virus or bacteria) detected in the patient.
"@en;
        d2rq:column "somatic15.Infectious_agent";
                d2rq:condition "somatic15.Infectious_agent != ('NA')";
        d2rq:condition "somatic15.Infectious_agent != ('')";
        d2rq:condition "somatic15.Infectious_agent != ('-')";
        .




# From Table somatic15  to sample details
map:sample a d2rq:ClassMap;
        d2rq:dataStorage map:database;
        d2rq:uriPattern "sample/@@somatic15.Sample_ID@@";
        d2rq:class logvd:sample;
        d2rq:classDefinitionLabel "sample details";
        d2rq:classDefinitionComment " Tumor site, type and origin.   Tumor samples are
classified according to standards of the International Classification of Diseases for Oncology
(ICD-O 3rd Edition, World Health Organization, Geneva, 2000) and SNOMED. "@en;
        .
map:sample__label a d2rq:PropertyBridge;
        d2rq:belongsToClassMap map:sample;
        d2rq:property rdfs:label;
        d2rq:pattern "sample/@@somatic15.Sample_Name@@";
        .


map:sample_Sample_Name a d2rq:PropertyBridge;
        d2rq:belongsToClassMap map:sample;
        d2rq:property logvd:Name;
        d2rq:propertyDefinitionLabel "sample Sample Name";
        d2rq:propertyDefinitionComment " Name or number given by the authors to the tumor sample
or cell line. If the name is not distinctive, e.g., if the publication refers to samples as
tumors 1,2,3, etc, we have arbitrarily assigned a name, usually the first letters of the first
author's name, followed by the numbers in the series. The same name or number can occur several
times as in some samples more than one mutation has been reported. "@en;
        d2rq:column "somatic15.Sample_Name";
                d2rq:condition "somatic15.Sample_Name != ('NA')";
        d2rq:condition "somatic15.Sample_Name != ('')";
        d2rq:condition "somatic15.Sample_Name != ('-')";
        .
map:sample_Sample_source a d2rq:PropertyBridge;
        d2rq:belongsToClassMap map:sample;
        d2rq:property logvd:Sample_source;
        d2rq:propertyDefinitionLabel "sample Sample source";
        d2rq:propertyDefinitionComment " Nature of the sample from which the mutation has been
identified: cell-line, surgery (surgical or autopsy specimen, including fresh samples and
archival, pathology specimen), biopsy, xenograft, body fluid (blood, saliva, urine...). "@en;
        d2rq:column "somatic15.Sample_source";
                d2rq:condition "somatic15.Sample_source != ('NA')";
        d2rq:condition "somatic15.Sample_source != ('')";
```

```
        d2rq:condition "somatic15.Sample_source != ('-')";
        .
map:sample_Tumor_origin a d2rq:PropertyBridge;
        d2rq:belongsToClassMap map:sample;
        d2rq:property logvd:Tumor_origin;
        d2rq:propertyDefinitionLabel "sample Tumor origin";
        d2rq:propertyDefinitionComment " Origin of the tumor sample. Terms occurring in this
column are: primary, secondary (second primary tumor in the same patient), metastasis (with the
localisation of the metastasis in brackets), recurrent (tumor recurrence). "@en;
        d2rq:column "somatic15.Tumor_origin";
                d2rq:condition "somatic15.Tumor_origin != ('NA')";
        d2rq:condition "somatic15.Tumor_origin != ('')";
        d2rq:condition "somatic15.Tumor_origin != ('-')";
        .
map:sample_Topography a d2rq:PropertyBridge;
        d2rq:belongsToClassMap map:sample;
        d2rq:property NCIT:Topography;
        d2rq:propertyDefinitionLabel "sample Topography";
        d2rq:propertyDefinitionComment " Site of the tumor defined by organ or group of organs,
according to the ICD-O nomenclature. "@en;
        d2rq:column "somatic15.Topography";
                d2rq:condition "somatic15.Topography != ('NA')";
        d2rq:condition "somatic15.Topography != ('')";
        d2rq:condition "somatic15.Topography != ('-')";
        .

map:sample_Topo_code a d2rq:PropertyBridge;
        d2rq:belongsToClassMap map:sample;
        d2rq:property logvd:Topo_code;
        d2rq:propertyDefinitionLabel "sample Topology code";
        d2rq:propertyDefinitionComment " ICD-O code for topography.  "@en;
        d2rq:column "somatic15.Topo_code";
                d2rq:condition "somatic15.Topo_code != ('NA')";
        d2rq:condition "somatic15.Topo_code != ('')";
        d2rq:condition "somatic15.Topo_code != ('-')";
        .
map:sample_Sub_topography a d2rq:PropertyBridge;
        d2rq:belongsToClassMap map:sample;
        d2rq:property logvd:Sub_topography;
        d2rq:propertyDefinitionLabel "sample Sub topography";
        d2rq:propertyDefinitionComment " Precise identification of anatomic site, organ or
tissue. The description given in the publication is translated to ICD-O nomenclature. "@en;
        d2rq:column "somatic15.Sub_topography";
                d2rq:condition "somatic15.Sub_topography != ('NA')";
        d2rq:condition "somatic15.Sub_topography != ('')";
        d2rq:condition "somatic15.Sub_topography != ('-')";
        .
map:sample_Morphology a d2rq:PropertyBridge;
        d2rq:belongsToClassMap map:sample;
        d2rq:property NCIT:Neoplasm_by_Morphology;
        d2rq:propertyDefinitionLabel "sample Morphology";
        d2rq:propertyDefinitionComment " Tumor type, including morphology and-or histologic
type. The terminology used is based on ICD-O (2nd and 3rd editions) and SNOMED classifications.
Terms have been added, such as normal-tissue or na. "@en;
        d2rq:column "somatic15.Morphology";
                d2rq:condition "somatic15.Morphology != ('NA')";
        d2rq:condition "somatic15.Morphology != ('')";
        d2rq:condition "somatic15.Morphology != ('-')";
        .
map:sample_Morpho_code a d2rq:PropertyBridge;
        d2rq:belongsToClassMap map:sample;
        d2rq:property logvd:Morpho_code;
        d2rq:propertyDefinitionLabel "sample Morphology code";
        d2rq:propertyDefinitionComment " ICD-O or SNOMED codes for morphology. "@en;
        d2rq:column "somatic15.Morpho_code";
                d2rq:condition "somatic15.Morpho_code != ('NA')";
        d2rq:condition "somatic15.Morpho_code != ('')";
        d2rq:condition "somatic15.Morpho_code != ('-')";
        .
map:sample_Grade a d2rq:PropertyBridge;
        d2rq:belongsToClassMap map:sample;
        d2rq:property logvd:Grade;
        d2rq:propertyDefinitionLabel "sample tumor grade";
        d2rq:propertyDefinitionComment " Information on tumor grade, as given in the cited
publication. "@en;
        d2rq:column "somatic15.Grade";
                d2rq:condition "somatic15.Grade != ('NA')";
        d2rq:condition "somatic15.Grade != ('')";
```

```
        d2rq:condition "somatic15.Grade != ('-')";
        .
map:sample_Stage a d2rq:PropertyBridge;
        d2rq:belongsToClassMap map:sample;
        d2rq:property NCIT:Stage;
        d2rq:propertyDefinitionLabel "sample tumor stage";
        d2rq:propertyDefinitionComment " Information on tumor stage, as given in the cited
publication. "@en;
        d2rq:column "somatic15.Stage";
                d2rq:condition "somatic15.Stage != ('NA')";
        d2rq:condition "somatic15.Stage != ('')";
        d2rq:condition "somatic15.Stage != ('-')";
        .
map:sample_TNM a d2rq:PropertyBridge;
        d2rq:belongsToClassMap map:sample;
        d2rq:property NCIT:TNM;
        d2rq:propertyDefinitionLabel "sample TNM status";
        d2rq:propertyDefinitionComment "Tumor, Node and Metastasis status (TNM nomenclature).
"@en;
        d2rq:column "somatic15.TNM";
                d2rq:condition "somatic15.TNM != ('NA')";
        d2rq:condition "somatic15.TNM != ('')";
        d2rq:condition "somatic15.TNM != ('-')";
        .
map:sample_p53_IHC a d2rq:PropertyBridge;
        d2rq:belongsToClassMap map:sample;
        d2rq:property logvd:p53_IHC;
        d2rq:propertyDefinitionLabel "sample p53 IHC grade";
        d2rq:propertyDefinitionComment " p53 immunostaining grade. "@en;
        d2rq:column "somatic15.p53_IHC";
                d2rq:condition "somatic15.p53_IHC != ('NA')";
        d2rq:condition "somatic15.p53_IHC != ('')";
        d2rq:condition "somatic15.p53_IHC != ('-')";
        .
map:sample_Add_Info a d2rq:PropertyBridge;
        d2rq:belongsToClassMap map:sample;
        d2rq:property logvd:Add_Info;
        d2rq:propertyDefinitionLabel "sample Additional Information";
        d2rq:propertyDefinitionComment " Any useful additional information is entered here.
"@en;
        d2rq:column "somatic15.Add_Info";
                d2rq:condition "somatic15.Add_Info != ('NA')";
        d2rq:condition "somatic15.Add_Info != ('')";
        d2rq:condition "somatic15.Add_Info != ('-')";
        .


map:sample_Tissue_processing a d2rq:PropertyBridge;
        d2rq:belongsToClassMap map:sample;
        d2rq:property logvd:Tissue_processing;
        d2rq:propertyDefinitionLabel "Samples tissue processing";
        d2rq:propertyDefinitionComment "Indicates if the sample analysed was fresh, fixed or
frozen."@en;
        d2rq:join "somatic15.Ref_ID => SomaticRef15.Ref_ID";
        d2rq:column "SomaticRef15.Tissue_processing";
                d2rq:condition "SomaticRef15.Tissue_processing != ('NA')";
        d2rq:condition "SomaticRef15.Tissue_processing != ('')";
        d2rq:condition "SomaticRef15.Tissue_processing != ('-')";
        .
map:sample_Start_material a d2rq:PropertyBridge;
        d2rq:belongsToClassMap map:sample;
        d2rq:property logvd:Start_material;
        d2rq:propertyDefinitionLabel "Samples start material";
        d2rq:propertyDefinitionComment " Indicates if DNA or RNA was used to search for
mutations. "@en;
        d2rq:column "SomaticRef15.Start_material";
                d2rq:condition "SomaticRef15.Start_material != ('NA')";
        d2rq:join "somatic15.Ref_ID => SomaticRef15.Ref_ID";
        d2rq:condition "SomaticRef15.Start_material != ('')";
        d2rq:condition "SomaticRef15.Start_material != ('-')";
        .
map:sample_Prescreening a d2rq:PropertyBridge;
        d2rq:belongsToClassMap map:sample;
        d2rq:property logvd:Prescreening;
        d2rq:propertyDefinitionLabel "Samples Prescreening";
        d2rq:propertyDefinitionComment " Method used to detect the mutations. "@en;
        d2rq:join "somatic15.Ref_ID => SomaticRef15.Ref_ID";
        d2rq:column "SomaticRef15.Prescreening";
```

```
            d2rq:condition "SomaticRef15.Prescreening != ('NA')";
        d2rq:condition "SomaticRef15.Prescreening != ('')";
        d2rq:condition "SomaticRef15.Prescreening != ('-')";
        .
map:sample_Material_sequenced a d2rq:PropertyBridge;
        d2rq:belongsToClassMap map:sample;
        d2rq:property logvd:Material_sequenced;
        d2rq:propertyDefinitionLabel "Samples material sequenced";
        d2rq:propertyDefinitionComment " Indicates if the DNA or RNA was cloned or not before
sequencing. "@en;
        d2rq:join "somatic15.Ref_ID => SomaticRef15.Ref_ID";
        d2rq:column "SomaticRef15.Material_sequenced";
            d2rq:condition "SomaticRef15.Material_sequenced != ('NA')";
        d2rq:condition "SomaticRef15.Material_sequenced != ('')";
        d2rq:condition "SomaticRef15.Material_sequenced != ('-')";
        .

map:sample_Individual_ID a d2rq:PropertyBridge;
        d2rq:belongsToClassMap map:sample;
        d2rq:property logvd:hasIndividual;
        d2rq:propertyDefinitionLabel "somatic Individual ref";
        d2rq:propertyDefinitionComment " Unique identification number for an individual included
in the database. "@en;
        d2rq:refersToClassMap map:individual;
    d2rq:join "somatic15.Individual_ID => somatic15.Individual_ID";
        .




# IARC-TP53 the database

map:DB_source a d2rq:ClassMap;
        d2rq:dataStorage map:database;
        d2rq:uriPattern "database/IARC-TP53";
        d2rq:class logvd:IARC-TP53;
        d2rq:classDefinitionLabel "Database source";
        d2rq:classDefinitionComment "RDFized Database"@en;
        .
map:DB_source__label a d2rq:PropertyBridge;
        d2rq:belongsToClassMap map:DB_source;
        d2rq:property rdfs:label;
        d2rq:pattern "database/IARC-TP53";
        .


map:DB_source_database a d2rq:PropertyBridge;
        d2rq:belongsToClassMap map:DB_source;
        d2rq:property logvd:IARC_database;
        d2rq:propertyDefinitionLabel "IARC TP53 Somatic Mutation database (IARCDB)";
        d2rq:propertyDefinitionComment "IARC TP53 Somatic Mutation database (IARCDB)"@en;
        d2rq:column "iarc.DB";
        .

map:DB_source_DBPedia a d2rq:PropertyBridge;
        d2rq:belongsToClassMap map:DB_source;
        d2rq:property owl:sameAs;
        d2rq:propertyDefinitionLabel "IARC at DBPedia";
        d2rq:propertyDefinitionComment "IARC DBPedia resource"@en;
        d2rq:uriPattern
"http://dbpedia.org/resource/International_Agency_for_Research_on_Cancer";
        .

map:DB_source_current_release a d2rq:PropertyBridge;
        d2rq:belongsToClassMap map:DB_source;
        d2rq:property logvd:IARC_release;
        d2rq:propertyDefinitionLabel "IARC TP53 Somatic Mutation database current release";
        d2rq:propertyDefinitionComment "The release of the IARC TP53 Somatic Mutation database
currently deployed"@en;
        d2rq:column "iarc.rel";
        .

map:DB_source_DB_type a d2rq:PropertyBridge;
        d2rq:belongsToClassMap map:DB_source;
        d2rq:property logvd:DB_type;
        d2rq:propertyDefinitionLabel "IARC TP53 DataBase type";
```

```
        d2rq:propertyDefinitionComment "The typology of the IARC TP53 Somatic Mutation database
: Locus Specific DataBase"@en;
        d2rq:column "iarc.type";
        .

map:DB_source_target_gene a d2rq:PropertyBridge;
        d2rq:belongsToClassMap map:DB_source;
        d2rq:property logvd:hastarget_gene;
        d2rq:propertyDefinitionLabel "IARC database target gene";
        d2rq:propertyDefinitionComment "The gene whose information are collected at the IARC
TP53 Somatic Mutation database"@en;
        d2rq:uriPattern "gene/TP53";
        .

map:DB_source_URL a d2rq:PropertyBridge;
        d2rq:belongsToClassMap map:DB_source;
        d2rq:property logvd:IARC_URL;
        d2rq:propertyDefinitionLabel "IARC web address";
        d2rq:propertyDefinitionComment "Web address of the IARC database"@en;
        d2rq:uriPattern "http://www-p53.iarc.fr/";
        .

map:DB_source_view_coding-seq a d2rq:PropertyBridge;
        d2rq:belongsToClassMap map:DB_source;
        d2rq:property logvd:IARC_TP53_coding-seq;
        d2rq:propertyDefinitionLabel "IARC coding sequence";
        d2rq:propertyDefinitionComment "View coding sequence with residue numbers, amino-acids,
highlighted CpG sites and exon-intron boundaries."@en;
        d2rq:uriPattern "http://www-p53.iarc.fr/p53Sequence.html";
        .

map:DB_source_view_refseq a d2rq:PropertyBridge;
        d2rq:belongsToClassMap map:DB_source;
        d2rq:property logvd:IARC_TP53_refseq;
        d2rq:propertyDefinitionLabel "IARC reference sequence";
        d2rq:propertyDefinitionComment "View NC_000017.9 gene sequence (new reference sequence
used from R13 version) with exon-intron boundaries and highlighted polymorphic sites."@en;
        d2rq:uriPattern "http://www-p53.iarc.fr/TP53sequence_NC_000017-9.html";
        .


map:DB_source_somatic_mutation a d2rq:PropertyBridge;
        d2rq:belongsToClassMap map:DB_source;
        d2rq:property logvd:TP53_somatic_mutation;
        d2rq:propertyDefinitionLabel "TP53 somatic mutations";
        d2rq:propertyDefinitionComment "IARC TP53 somatic mutations reported in the
database."@en;
        d2rq:uriPattern "somatic_mutation/@@somatic15.Mutation_ID@@";
        .





# IARC-TP53 the database

map:gene a d2rq:ClassMap;
        d2rq:dataStorage map:database;
        d2rq:uriPattern "gene/TP53";
        d2rq:class logvd:gene;
        d2rq:classDefinitionLabel "Genes";
        d2rq:classDefinitionComment "Genes"@en;
        .
map:gene__label a d2rq:PropertyBridge;
        d2rq:belongsToClassMap map:gene;
        d2rq:property rdfs:label;
        d2rq:pattern "gene/TP53";
        .

map:gene_ENSEMBL_ID a d2rq:PropertyBridge;
        d2rq:belongsToClassMap map:gene;
        d2rq:property rdfs:seeAlso;
        d2rq:propertyDefinitionLabel "TP53 ENSEMBL gene view";
        d2rq:propertyDefinitionComment "TP53 gene view at the ENSEMBL database."@en;
        d2rq:uriPattern "http://www.ensembl.org/Homo_sapiens/geneview?db=&gene=ENSG00000141510";
        .
```

```
map:gene_OMIM a d2rq:PropertyBridge;
        d2rq:belongsToClassMap map:gene;
        d2rq:property owl:sameAs;
        d2rq:propertyDefinitionLabel "TP53 gene OMIM ref";
        d2rq:propertyDefinitionComment "TP53 gene ref at the OMIM database."@en;
        d2rq:uriPattern "http://bio2rdf.org/omim:191170";
        .

map:gene_DBPedia_link a d2rq:PropertyBridge;
        d2rq:belongsToClassMap map:gene;
        d2rq:property rdfs:seeAlso;
        d2rq:propertyDefinitionLabel "P53 DBPedia link";
        d2rq:propertyDefinitionComment "DBPedia link to the P53 resource"@en;
        d2rq:uriPattern "http://dbpedia.org/resource/P53";
        .

map:gene_HGNC a d2rq:PropertyBridge;
        d2rq:belongsToClassMap map:gene;
        d2rq:property diseasome:hgncId;
        d2rq:propertyDefinitionLabel "HGNC TP53 Gene Symbol ref";
        d2rq:propertyDefinitionComment "HGNC TP53 Gene Symbol ID"@en;
        d2rq:uriPattern "http://bio2rdf.org/hgnc:11998";
        .


map:gene_gene a d2rq:PropertyBridge;
        d2rq:belongsToClassMap map:gene;
        d2rq:property diseasome:geneId;
        d2rq:propertyDefinitionLabel "TP53 Entrez Gene";
        d2rq:propertyDefinitionComment "TP53 gene at the NCBI database."@en;
        d2rq:uriPattern "http://bio2rdf.org/geneid:7157";
        .
map:gene_gene_product a d2rq:PropertyBridge;
        d2rq:belongsToClassMap map:gene;
        d2rq:property logvd:hasproduct;
        d2rq:propertyDefinitionLabel "TP53 gene product";
        d2rq:propertyDefinitionComment "TP53 gene product at the UniProt database."@en;
        d2rq:uriPattern "http://bio2rdf.org/uniprot:P04637";
        .

map:gene_gene_variation a d2rq:PropertyBridge;
        d2rq:belongsToClassMap map:gene;
        d2rq:property logvd:hasgene_variation;
        d2rq:propertyDefinitionLabel "TP53 gene variations";
        d2rq:propertyDefinitionComment "TP53 gene variations reported in the database."@en;
        d2rq:uriPattern "variation/@@mutations15.Mut_ID@@";
        .
```
